# Effects of postoperative complications in oesophageal cancer on survival, hospital outcomes, and long-term quality of life: retrospective cohort study

**DOI:** 10.1093/bjsopen/zraf083

**Published:** 2025-08-02

**Authors:** Nerma Crnovrsanin, Stefan Giring, Antonia Oppel, Ingmar F Rompen, Sabine Schiefer, Nicolas Jorek, Thomas Schmidt, Beat P Müller-Stich, Leila Sisic, Henrik Nienhüser

**Affiliations:** Department of General, Abdominal and Transplantation Surgery, University Hospital Heidelberg, Heidelberg, Germany; Department of General, Abdominal and Transplantation Surgery, University Hospital Heidelberg, Heidelberg, Germany; Department of General, Abdominal and Transplantation Surgery, University Hospital Heidelberg, Heidelberg, Germany; Department of General, Abdominal and Transplantation Surgery, University Hospital Heidelberg, Heidelberg, Germany; Department of General, Abdominal and Transplantation Surgery, University Hospital Heidelberg, Heidelberg, Germany; Department of General, Abdominal and Transplantation Surgery, University Hospital Heidelberg, Heidelberg, Germany; Department of General, Abdominal, Thoracic and Transplantation Surgery, University Hospital Cologne, Cologne, Germany; Department of Surgery, Clarunis University Center for Gastrointestinal and Liver Disease, University Hospital and St. Clare Hospital Basel, Basel, Switzerland; Department of General, Abdominal and Transplantation Surgery, University Hospital Heidelberg, Heidelberg, Germany; Department of General, Abdominal and Transplantation Surgery, University Hospital Heidelberg, Heidelberg, Germany

## Abstract

**Introduction:**

Postoperative complications pose a major challenge in oesophageal surgery, affecting survival, recovery, and healthcare resource utilization. The aim of this study was to quantify the proportional contribution of specific complications to survival and adverse outcomes and to evaluate their effects on long-term quality of life (QoL) in patients with oesophageal and gastro-oesophageal junction cancer.

**Methods:**

This retrospective cohort study included patients with oesophageal or gastro-oesophageal junction cancer who underwent surgery with curative intent between January 2010 and July 2022. Postoperative complications were categorized following Esophageal Complications Consensus Group guidelines. Population-attributable fractions (PAFs) were calculated to estimate the proportion of adverse outcomes and survival effects theoretically preventable if specific complications were avoided.

**Results:**

In 632 patients who underwent surgery, the most frequently observed complications were pulmonary (31%), infectious (29%), and gastrointestinal (24%). Pneumonia had the highest adjusted PAF for overall survival (8.3% after 2 years; 95% confidence interval (c.i.) 1.8 to 14.7), suggesting that preventing pneumonia could substantially reduce mortality. Anastomotic leak had the highest PAF for recurrence-free survival (6.6%; 95% c.i. 1.8 to 11.5) and was the complication most significantly contributing to reoperations (PAF 39.8%; 95% c.i. 22.2 to 52.1) and prolonged hospital stays (PAF 56.9%; 95% c.i. 46.8 to 66.2). Respiratory failure had the largest effect on 90-day mortality (PAF 53.5%; 95% c.i. 30.9 to 73.9). In contrast, no significant effect of complications on long-term QoL was observed.

**Conclusion:**

This study underscores the critical importance of targeted strategies to prevent postoperative complications, particularly pneumonia and anastomotic leakage, which contribute significantly to adverse outcomes such as reduced survival and prolonged hospital stays. Effective complication management may enhance oncological outcomes and optimize healthcare resource utilization.

## Teaser

This study evaluated the impact of postoperative complications on long-term survival and recurrence in 632 patients with oesophageal cancer using population-attributable fractions (PAFs) and multivariable Cox regression. Pneumonia had the highest adjusted PAF for overall survival (8.3% at 2 years), whereas anastomotic leak significantly affected recurrence-free survival (PAF 6.6%) and hospital outcomes. These findings emphasize the proportional contribution of complications to survival and the need for targeted perioperative strategies.

## Introduction

Postoperative complications remain a relevant risk in surgery because they lead to prolonged recovery, increased morbidity, and higher healthcare costs, even in the most carefully performed procedures^1^. Particularly in the treatment of oesophageal cancer, complication rates remain high and their consequences severe, despite significant improvements and reductions in morbidity and mortality over the past 25 years^2^. The reported prevalence of complications in oesophageal cancer patients varies in the literature. Recent studies focusing on patients treated over the past decade report complication rates ranging from 59 to 76%, with pulmonary complications, cardiac complications, and anastomotic leaks being the most common^3–5^.

Postoperative complications can influence multiple postoperative adverse outcomes, such as 90-day mortality^6^, reoperation, and prolonged hospital stay, as well as survival outcomes^3,7^, quality of life, costs, and resource utilization^8^. Furthermore, patients with complications are often not able to receive postoperative therapy, indirectly influencing long-term survival^9^. Therefore, there is a high clinical need to better understand the impact of postoperative complications on outcome. In particular, identifying complications that have the most impact on adverse and survival outcomes can help prioritize preventive actions and quality improvement programs to reduce their clinical and economic burden^10,11^.

The aim of the present study was to make a broad assessment of postoperative complications and to assess which of them have the most impact on clinical adverse and survival outcomes after the introduction of multimodal treatment strategies using the standardized list of complications proposed by the Esophagectomy Complications Consensus Group (ECCG).^12^

## Methods

### Study population and clinicopathological information

Patients from a prospectively maintained database who had been treated at the Department of General Surgery, University Hospital Heidelberg between January 2010 and July 2022 were analysed. Patients who had adenocarcinoma or squamous cell carcinoma (SCC) of the oesophagus or gastro-oesophageal junction and who underwent surgery with curative intent with either a transhiatal gastrectomy or thoracoabdominal oesophageal resection were included in the study.

Patients received standard of care therapy according to contemporary and current guidelines in consensus following multidisciplinary team discussion^13^. Patients with distant metastases (cM1) and oligometastatic disease were included if they underwent surgery with curative intent based on individual treatment decisions, provided that the metastatic lesions had either a complete response to preoperative chemotherapy or were resected alongside the primary tumour^14^. Clinicopathological and follow-up data were collected prospectively and analysed retrospectively.

Informed consent was obtained from all patients and the study was approved by the institutional ethics committee of the Heidelberg University (S-635-2013).

The American Society of Anesthesiologists (ASA) physical status classification system was used to assess medical co-morbidities and perioperative risks by experienced anaesthesiologists and surgeons^15^. Severe co-morbidities were defined as decompensated renal insufficiency, decompensated cardiac insufficiency, liver cirrhosis, status post (s/p) myocardial infarction, s/p valve replacement, s/p stroke, s/p carotid stenosis, severe coronary heart disease, complicated diabetes, chronic pancreatitis, chronic obstructive pulmonary disease, or lung emphysema^16^. The histopathological work-up and response assessment were classified and staged according to the recommendations of the Union for International Cancer Control, 8th edition^17^. Histopathological response to neoadjuvant chemotherapy was graded according to Becker *et al*.^18^.

Patients were followed up on an outpatient basis by the Medical Oncology Department, University Hospital Heidelberg according to a standardized protocol or by other treating physicians, as described previously^19^. The last follow-up was on 31 August 2024.

### Definitions of complications and outcomes

All complications were evaluated and classified according to the definitions proposed by the ECCG^12^ and graded according to the Clavien–Dindo (CD) classification^20^. In addition, the classification was extended to include the complications of enterothorax, pylorospasm, and delayed gastric emptying. All events within 30 days after surgery were included as postoperative complications. Complications were categorized according to their highest CD grade.

The primary outcome measures were as follows: overall survival (OS), defined as the time from diagnosis until death or last follow-up; and recurrence-free survival (RFS), defined as the time from surgery until the recurrence of disease. Secondary outcomes included reoperation (defined as surgical intervention under general anaesthesia), prolonged hospital stay (defined as a length of hospital stay equal or greater than the 75th percentile and stratified for surgical access (open *versus* laparoscopic/robotic)), 90-day mortality, and long-term health-related quality of life (HRQoL), based on the European Organization for Research and Treatment of Cancer (EORTC) QLQ-C30 and QLQ-OG25 questionnaires^22^. Because the purpose of this study was to analyse the effect of complications on long-term oncological outcomes, patients who died during admission or within 90 days of surgery for survival analysis were excluded^21^.

### HRQoL assessment

Information on long-term HRQoL was obtained from a previously conducted study, in which HRQoL was assessed at a single time point at least 2 years after surgery to evaluate the presence of lasting symptoms after surgeries for upper gastrointestinal cancers. Only patients who were alive at least 2 years after surgery were contacted for this assessment. HRQoL was assessed using the validated the EORTC QLQ-C30 and tumour-specific QLQ-OG25 questionnaires^22^, and the analysis was performed according to the recommendations of the SISAQOL Consortium^23^.

### Statistical analysis

Continuous variables are presented as the median with interquartile range (IQR), and categorical variables are reported as numbers and percentages. Categorical variables were compared using χ^2^ tests and Fisher’s exact tests, whereas continuous variables were compared using the Mann–Whitney *U* test or Kruskal–Wallis test. Pairwise correlations between binary postoperative complications were assessed using Pearson's correlation coefficient. All tests were two-sided and *P* < 0.050 was considered statistically significant.

Complications that did not have 20 events were not considered for statistical analysis. Survival rates were estimated using the Kaplan–Meier method. The significance of differences in survival among groups was calculated using the log-rank test. A Cox proportional hazard regression was performed and adjusted for the following variables based on previous literature and expert consensus: age, postoperative N and M stage, R stage, ASA classification, severe co-morbidities, type of surgery, perioperative treatment and histopathological subtype. A conditional OS analysis was also conducted after 1 year.^24^ For classes of complications significantly associated with OS and/or RFS, the adjusted population-attributable fraction (PAF) was calculated separately^25^.

The adjusted PAF estimates the expected percentage reduction in the adverse outcome if a specific complication were completely prevented in the study population, and is traditionally used in the epidemiological literature^25^. This measure quantifies the proportion of an outcome (for example, reoperation) attributable to a specific risk factor (for example, anastomotic leak), estimating the potential percentage reduction in the outcome if the risk factor were eliminated. The advantage of using the PAF lies in its ability to account for both the frequency and relative risk (RR) or hazard ratio (HR) of complications^10,11,26,27^.

For secondary outcomes (excluding quality of life (QoL)), the adjusted RR with a 95% confidence interval (c.i.) for each complication–outcome pair was calculated using a Poisson regression model with log link and robust error variance^28^. The adjusted PAF was estimated separately for each significant complication–outcome pair^28^. The confounders included were age, ASA classification, body mass index, severe co-morbidities, type of surgery, and surgical access (open *versus* laparoscopic/robotic), and were based on previous literature and expert consensus^29^.

In the comparison of QoL parameters, a difference higher than 10 points in the median scores between the group with and without a complication and a significant result in the hypothesis testing were deemed clinically relevant^30^.

Figures were generated and analyses were performed using R version 4.3.1 (R Foundation for Statistical Computing, Vienna, Austria) and the AF package (v0.1.5)^26^, among tidyverse (v2.0.0) and gtsummary (v1.7.2)^31,32^.

## Results

### Baseline characteristics

In all, 632 patients were included in the analysis. The median age was 63 (i.q.r. 56–69) years. Thoracoabdominal resection was the most common procedure (78%) and a gastric pull-up (73%) the most common type of reconstruction. Of the 632 patients, 239 (38%) had no complications (CD grade 0) and 21 (3.3%) died as a result of a postoperative complication (CD grade V). Examination of preoperative variables revealed that patients without any complication had a significantly lower ASA classification (*P* = 0.006) and less severe co-morbidities (*P* = 0.020). An overview of the comparison between patients with and without complications, as well as the clinicopathological characteristics of the entire cohort, is presented in *Table 1*.

**Table 1:** Clinicopathological characteristics of the entire study population, as well as patients with and without complications separately

	Overall (*n* = 632)	No complications (*n* = 239)	Complications (*n* = 393)	P*
Age (years), median (i.q.r.)	63 (56–69)	62 (56–69)	63 (56–70)	0.159
**ASA grade**				0.006†
I/II	261 (42%)	115 (49%)	146 (38%)	
III/IV	362 (58%)	120 (51%)	242 (62%)	
BMI (kg/m^2^), median (i.q.r.)	25.7 (23.5–28.7)	25.7 (23.4–28.7)	25.7 (23.5–28.5)	0.901
**Sex**				0.813†
Female	122 (19%)	45 (19%)	77 (20%)	
Male	510 (81%)	194 (81%)	316 (80%)	
Severe co-morbidities	205 (33%)	64 (27%)	141 (36%)	0.020†
Cardiac co-morbidities	320 (51%)	117 (49%)	203 (52%)	0.557†
Pulmonary co-morbidities	102 (16%)	30 (13%)	72 (18%)	0.060†
Metabolic co-morbidities	136 (22%)	47 (20%)	89 (23%)	0.396†
**Histopathological subtype**				0.358†
Adenocarcinoma	534 (84%)	206 (86%)	328 (83%)	
SCC	98 (16%)	33 (14%)	65 (17%)	
**Tumour stage**				
cT				0.116†
I/II	91 (16%)	32 (15%)	59 (17%)	
III	423 (76%)	170 (80%)	253 (73%)	
IV	44 (7.9%)	11 (5.2%)	33 (9.6%)	
cN				0.850†
cN0	128 (21%)	48 (21%)	80 (21%)	
cN1–3	479 (79%)	184 (79%)	295 (79%)	
cM				0.255†
cM0	566 (91%)	213 (89%)	353 (92%)	
cM1	55 (8.9%)	25 (11%)	30 (7.8%)	
**Type of NAC**				0.985†
None	112 (18%)	43 (18%)	69 (18%)	
Epirubicin-based	61 (10%)	24 (10%)	37 (9.8%)	
FLOT/FLO	343 (56%)	132 (57%)	211 (56%)	
RCTx	93 (15%)	34 (15%)	59 (16%)	
Interruption of NAC	41 (8.2%)	13 (6.8%)	28 (9.1%)	0.361†
**pT**				0.317†
pT0	99 (16%)	42 (18%)	57 (15%)	
pTI/II	198 (31%)	67 (28%)	131 (34%)	
pTIII	312 (50%)	120 (50%)	192 (49%)	
pTIVa/b	20 (3.2%)	10 (4.2%)	10 (2.6%)	
**pN**				0.234†
pN0	322 (51%)	115 (48%)	207 (53%)	
pN1–3	305 (49%)	123 (52%)	182 (47%)	
Lymph node ratio	0.00 (0.00–0.13)	0.03 (0.00–0.16)	0.00 (0.00–0.12)	0.245
**pM**				0.737†
pM0	600 (95%)	226 (95%)	374 (95%)	
pM1	32 (5.1%)	13 (5.4%)	19 (4.8%)	
**R grade**				0.483†
0	564 (90%)	216 (91%)	348 (89%)	
1	65 (10%)	22 (9.2%)	43 (11%)	
Complete pathological regression	193 (40%)	79 (44%)	114 (38%)	0.195†
Adjuvant treatment	256 (47%)	123 (59%)	133 (40%)	< 0.001†
**Type of surgery**				0.014†
Thoracoabdominal resection	493 (78%)	174 (73%)	319 (81%)	
Transhiatal gastrectomy	139 (22%)	65 (27%)	74 (19%)	
**Localization of anastomosis**				0.004†
Cervical	17 (2.7%)	2 (0.8%)	15 (3.8%)	
Intra-abdominal	53 (8.4%)	30 (13%)	23 (5.9%)	
Intrathoracic, mediastinum	97 (15%)	39 (16%)	58 (15%)	
Intrathoracic, level of azygos vein	464 (74%)	168 (70%)	296 (76%)	
**Type of reconstruction**				0.043†
Gastric pull-up	460 (73%)	162 (68%)	298 (76%)	
Roux-en-Y	141 (22%)	66 (28%)	75 (19%)	
Other	29 (4.6%)	10 (4.2%)	19 (4.8%)	
Laparoscopic surgery	122 (19%)	39 (16%)	83 (21%)	0.138†
Robotic surgery	58 (9.2%)	18 (7.5%)	40 (10%)	0.264†
Duration of surgery (min), median (i.q.r.)	292 (235–368)	284 (226–343)	301(240–387)	0.005
Length of ICU stay (days), median (i.q.r.)	6 (3–12)	4 (2–6)	9 (5–20)	< 0.001
Length of hospital stay (days), median (i.q.r.)	18 (14–30)	14 (12–17)	25(17–38)	< 0.001
Intraoperative complication	55 (8.8%)	13 (5.5%)	42 (11%)	0.020†
**Clavien–Dindo classification**				< 0.001‡
0	239 (38%)	239 (100%)	0 (0%)	
I	77 (12%)	0 (0%)	77 (20%)	
II	54 (8.5%)	0 (0%)	54 (14%)	
IIIa	66 (10%)	0 (0%)	66 (17%)	
IIIb	90 (14%)	0 (0%)	90 (23%)	
IVa	73 (12%)	0 (0%)	73 (19%)	
IVb	12 (1.9%)	0 (0%)	12 (3.1%)	
V	21 (3.3%)	0 (0%)	21 (5.3%)	
90-day mortality	31 (4.9%)	4 (1.7%)	27 (6.9%)	0.003‡

Values are *n* (%) unless otherwise stated. i.q.r., interquartile range; ASA, American Society of Anesthesiologists; BMI, body mass index; SCC, squamous cell carcinoma; NAC, neoadjuvant chemotherapy; FLOT, fluorouracil, leucovorin, oxaliplatin, and docetaxel; FLO, fluorouracil, leucovorin, and oxaliplatin; RCTx, radiochemotherapy; ICU, intensive care unit. *Wilcoxon rank-sum test, except †Pearson's χ^2^ test or ‡Fisher's exact test.

An overview of the complications of the study population is presented in *Table 2*. Pulmonary complications (31%) were the most prevalent, followed by infectious (29%) and gastrointestinal (24%) complications. In all, 107 patients (17%) underwent reoperation due to postoperative complications.

**Table 2:** Postoperative complications in the study cohort (n = 632)

Characteristic	No. of patients
Patients with complication	393 (62%)
Clavien–Dindo grade > IIIa	196 (31%)
**Pulmonary complications**	199 (31%)
Pneumonia	151 (24%)
Pleural effusion	77 (12%)
Pneumothorax	41 (6.5%)
Respiratory failure requiring reintubation	110 (17%)
Tracheobronchial injury	7 (1.1%)
**Cardiac complication**	109 (17%)
Cardiac arrest requiring CPR	18 (2.8%)
Myocardial infarction	12 (1.9%)
Dysrhythmia requiring treatment	68 (11%)
**Gastrointestinal complication**	153 (24%)
Anastomotic leak	141 (22%)
Conduit necrosis	18 (2.8%)
Ileus	5 (0.8%)
Pylorospasm	28 (4.4%)
Bleeding requiring intervention	26 (4.1%)
Delayed gastric emptying	46 (7.3%)
Pancreatitis	7 (1.1%)
**Infectious complication**	182 (29%)
Wound infection	44 (7.0%)
Intrathoracic/intra-abdominal abscess	97 (15%)
Generalized sepsis	52 (8.2%)
Urological complication	40 (6.3%)
Thromboembolic complication	34 (5.4%)
**Neurological/psychiatric complication**	46 (7.3%)
Vocal cord injury/palsy	8 (1.3%)
**Wound/diaphragm complications**	51 (8.1%)
Abdominal wall dehiscence/hernia	11 (1.7%)
Enterothorax	18 (2.8%)
**Other complication**	106 (17%)
Chyle leak	18 (2.8%)
Conservative management	336 (53%)
Interventional management	208 (33%)
Reoperation	107 (17%)

CPR, cardiopulmonary resuscitation.

### Impact of complications on prognosis

**Fig. 1:**
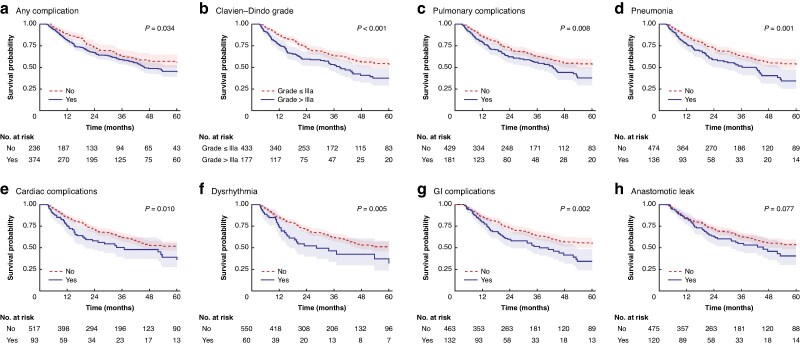
Kaplan–Meier curves of OS for patients with any complication, and for those with specific complications that were significantly associated with OS **a** Any complication, **b** Clavien–Dindo classification, **c** pulmonary complications, **d** pneumonia, **e** cardiac complications, **f** dysrhythmia, **g** GI complications, and **h** anastomotic leak. Shaded areas represent 95% confidence intervals. An overview of results from log-rank tests and the probabilities of 5-year OS and recurrence-free survival for specific complications are provided in *Table S1*. OS, overall survival; GI, gastrointestinal.

After excluding 37 patients (5.8%) who died either while in hospital or within the first 90 days after the procedure mortalities, an analysis of the association of complications with survival outcomes was performed in the remaining 595 patients. These 595 patients had a median follow-up of 41 (i.q.r. 38–46) months; during this time there were 221 (37.1%) deaths and 217 recurrences (36.5%). Kaplan–Meier estimates of the OS rate at 1, 3, and 5 years were 85% (95% c.i. 82 to 88), 61% (95% c.i. 57 to 66), and 50% (95% c.i. 46 to 56), respectively. The estimated RFS rates at 1, 3, and 5 years were 73% (95% c.i. 70 to 77), 57% (95% c.i. 53 to 62), and 51% (95% c.i. 46 to 57), respectively. An overview of the survival data and results from the log-rank test based on complication groups and specific complications is provided in *Table S1*, with Kaplan–Meier curves showing significant results in *Fig. 1*.

*Table 3* provides an overview of the adjusted HRs for OS and RFS. In general, complications associated requiring an intervention under general anaesthesia, classified as CD grade IIIb or higher (HR 1.68; 95% c.i. 1.23 to 2.30; *P* = 0.001), were associated with worse OS. Specifically, gastrointestinal complications (HR 1.75; 95% c.i. 1.25 to 2.44; *P* = 0.001), such as anastomotic leak (HR 1.56; 95% c.i. 1.11 to 2.20; *P* = 0.011), delayed gastric emptying (HR 1.76; 95% c.i. 1.04 to 2.98; *P* = 0.036), and conduit necrosis (HR 2.94; 95% c.i. 1.24 to 7.00; *P* = 0.014), and pulmonary complications (HR 1.62; 95% c.i. 1.16 to 2.25; *P* = 0.004), particularly pneumonia (HR 1.61; 95% c.i. 1.14 to 2.28; *P* = 0.007) and respiratory failure requiring reintubation (HR 1.70; 95% c.i. 1.15 to 2.53; *P* = 0.008), had significant negative effects on OS in the adjusted Cox regression analysis.

**Table 3:** Complications and adjusted hazard ratios for OS and RFS

	Reference group (no. without event)	No. with event	OS		RFS	
			aHR*	*P*	aHR*	*P*
Clavien–Dindo grade > 0	235	360	1.28 (0.95, 1.74)	0.110	1.02 (0.76, 1.36)	0.914
Clavien–Dindo grade > IIIa	425	170	1.68 (1.23, 2.30)	0.001	1.17 (0.85, 1.61)	0.349
**Pulmonary complication**	420	175	1.62 (1.16, 2.25)	0.004	1.34 (0.96, 1.87)	0.082
Pneumonia	465	130	1.61 (1.14, 2.28)	0.007	1.49 (1.05, 2.13)	0.027
Pleural effusion	526	69	1.06 (0.65, 1.74)	0.816	0.92 (0.57, 1.50)	0.745
Pneumothorax	557	38	0.85 (0.44, 1.66)	0.640	0.65 (0.32, 1.30)	0.223
Respiratory failure requiring reintubation	511	84	1.70 (1.15, 2.53)	0.008	1.25 (0.81, 1.93)	0.309
**Cardiac complication**	506	89	1.33 (0.89, 1.98)	0.160	1.19 (0.79, 1.78)	0.405
Dysrhythmia requiring treatment	538	57	1.32 (0.81, 2.16)	0.260	1.17 (0.69, 1.96)	0.561
**Gastrointestinal complication**	463	132	1.75 (1.25, 2.44)	0.001	1.69 (1.20, 2.37)	0.002
Anastomotic leak	475	120	1.56 (1.11, 2.20)	0.011	1.65 (1.16, 2.32)	0.005
Conduit necrosis	585	10	2.94 (1.24, 7.00)	0.014	2.21 (0.88, 5.54)	0.092
Pylorospasm	567	28	0.53 (0.20, 1.44)	0.213	0.43 (0.16, 1.18)	0.101
Bleeding requiring intervention	577	18	1.90 (0.83, 4.37)	0.128	1.13 (0.45, 2.85)	0.790
Delayed gastric emptying	550	45	1.76 (1.04, 2.98)	0.036	1.63 (0.97, 2.76)	0.067
**Infectious complication**	438	157	1.35 (0.96, 1.88)	0.081	1.33 (0.96, 1.85)	0.089
Wound infection	557	38	1.20 (0.69, 2.10)	0.520	1.33 (0.75, 2.36)	0.328
Intrathoracic/intra-abdominal abscess	508	87	1.22 (0.80, 1.86)	0.366	1.09 (0.69, 1.72)	0.702
Generalized sepsis	565	30	1.62 (0.90, 2.91)	0.107	1.91 (1.09, 3.36)	0.024
Urological complication	562	33	1.63 (0.90, 2.95)	0.107	1.20 (0.63, 2.28)	0.584
Thromboembolic complication	566	29	1.15 (0.56, 2.37)	0.709	0.97 (0.42, 2.22)	0.941
Neurological/psychiatric complication	555	40	1.07 (0.58, 1.98)	0.837	0.96 (0.53, 1.75)	0.899
Wound/diaphragm complication	550	45	1.16 (0.68, 1.99)	0.583	1.23 (0.71, 2.15)	0.461
Other complication	503	92	1.17 (0.76, 1.79)	0.472	0.88 (0.57, 1.38)	0.587

Values in parentheses are 95% confidence intervals. *Adjusted for age, (y)pN, (y)pM, R grade, American Society of Anesthesiologists classification, perioperative treatment, type of surgery and histological subtype. OS, overall survival; RFS, recurrence-free survival; aHR, adjusted hazard ratio.

Pneumonia (HR 1.49; 95% c.i. 1.05 to 2.13; *P* = 0.027), anastomotic leak (HR 1.65; 95% c.i. 1.16 to 2.32; *P* = 0.005), and generalized sepsis (HR 1.91; 95% c.i. 1.09 to 3.36; *P* = 0.024) were significantly associated with worse RFS.

For conditional multivariable analysis after 12 months (*Table 4*), 445 patients were eligible for analysis of OS. Although patients had survived for 1 year after surgery, in general, CD grade IIIb or higher complications were still associated with worse OS (HR 1.59; 95% c.i. 1.05 to 2.40; *P* = 0.027), as were pneumonia (HR 1.57; 95% c.i. 1.00 to 2.45; *P* = 0.050), respiratory failure (HR 1.75; 95% c.i. 1.03 to 2.97; *P* = 0.037), anastomotic leak (HR 2.06; 95% c.i. 1.34 to 3.15; *P* = 0.001), and delayed gastric emptying (HR 2.02; 95% c.i. 1.06 to 3.82; *P* = 0.032).

**Table 4:** Complications and adjusted hazard ratios for 1-year conditional overall survival

	Reference group (no. without event)	No. with event	1-Year aHR*	*P*
Clavien–Dindo grade > 0	186	259	1.14 (0.79, 1.66)	0.475
Clavien–Dindo grade > IIIa	333	112	1.59 (1.05, 2.40)	0.027
**Pulmonary complication**	327	118	1.51 (0.99, 2.33)	0.059
Pneumonia	357	88	1.57 (1.00, 2.45)	0.050
Pleural effusion	400	45	1.15 (0.61, 2.14)	0.669
Pneumothorax	420	25	0.62 (0.24, 1.56)	0.307
Respiratory failure requiring reintubation	391	54	1.75 (1.03, 2.97)	0.037
**Cardiac complication**	389	56	1.55 (0.94, 2.58)	0.088
Dysrhythmia requiring treatment	408	37	1.82 (0.99, 3.36)	0.054
**Gastrointestinal complication**	352	93	2.07 (1.36, 3.16)	0.001
Anastomotic leak	356	89	2.06 (1.34, 3.15)	0.001
Conduit necrosis	441	4	1.79 (0.41, 7.86)	0.438
Pylorospasm	425	20	0.39 (0.10, 1.59)	0.190
Bleeding requiring intervention	433	12	1.28 (0.31, 5.24)	0.735
Delayed gastric emptying	409	36	2.02 (1.06, 3.82)	0.032
**Infectious complication**	333	112	1.17 (0.76, 1.81)	0.467
Wound infection	418	27	0.94 (0.43, 2.06)	0.876
Intrathoracic/intra-abdominal abscess	386	59	1.32 (0.76, 2.27)	0.324
Generalized sepsis	428	17	1.71 (0.78, 3.79)	0.182
Urological complication	421	24	1.80 (0.87, 3.73)	0.115
Thromboembolic complication	423	22	1.19 (0.47, 3.00)	0.710
Neurological/psychiatric complication	420	25	1.02 (0.44, 2.36)	0.957
Wound/diaphragm complication	414	31	0.82 (0.37, 1.79)	0.614
Other complication	383	62	0.99 (0.57, 1.75)	0.984

Values in parentheses are 95% confidence intervals. *Adjusted for age, (y)pN, (y)pM, R grade, American Society of Anesthesiologists classification, perioperative treatment, type of surgery and histological subtype. aHR, adjusted hazard ratio.

### PAFs of postoperative complications and their effects on primary and secondary outcomes

The PAF estimates the proportion of poor survival outcomes that could theoretically be avoided if a particular complication were effectively managed or prevented.

**Fig. 2:**
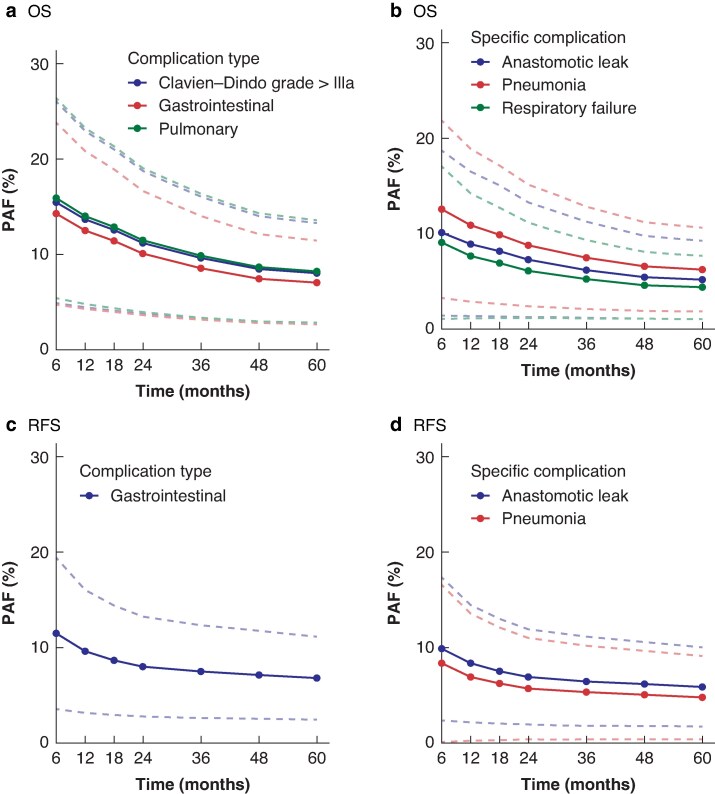
Significant risk-adjusted PAF estimates for OS and RFS PAF estimates for **a,b** OS and **c,d** RFS according to complication types and specific complications after adjustment for age, postoperative N and M stage, R grade, American Society of Anesthesiologists classification, severe co-morbidities, type of surgery, perioperative treatment, and histopathological subtype in a Cox regression model. Dashed lines represent 95% confidence intervals. PAF, population-attributable fraction; OS, overall survival; RFS, recurrence-free survival.

Compared with other complication types, pulmonary complications had the greatest impact on OS > 2 years after surgery, with a risk-adjusted PAF estimate of 10.9% (95% c.i. 3.2 to 18.7; *Fig. 2a*; *Table S2*). However, any complication classified as CD grade > IIIa showed a risk-adjusted PAF estimate for OS > 2 years after surgery of 11.7% (95% c.i. 4.1 to 19.4). Only gastrointestinal complications had a significant effect on RFS (*Fig. 2c*; *Table S2*). Among specific complications, postoperative pneumonia had the greatest impact on OS > 2 years after surgery, with a risk-adjusted PAF estimate of 8.3% (95% c.i. 1.8 to 14.7), and anastomotic leak had the greatest impact on RFS > 2 years after surgery, with a risk-adjusted PAF estimate of 6.6% (95% c.i. 1.8 to 11.5; *Fig. 2b,d*; *Table S3*).

**Fig. 3:**
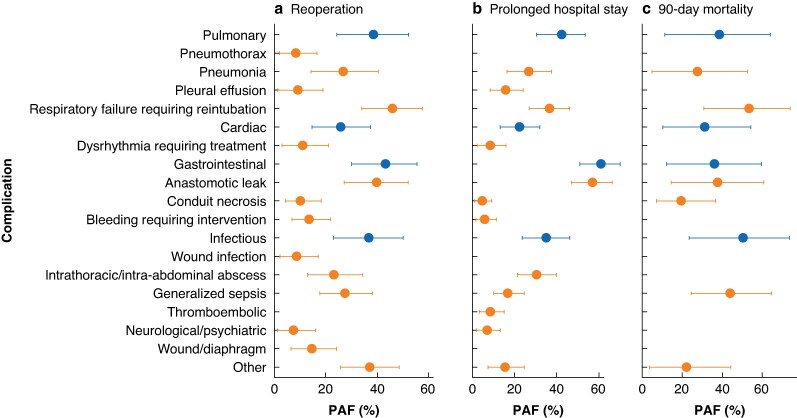
Significant risk-adjusted PAF estimates for in-hospital adverse outcomes PAF estimates for **a** reoperation, **b** prolonged hospital stay, and **c** 90-day mortality according to complication type (blue) and specific complications (orange) within each complication type after adjustment for age, American Society of Anesthesiologists classification, body mass index, severe co-morbidities, type of surgery, and surgical access (open *versus* laparoscopic/robotic) in a Poisson regression model. PAF, population-attributable fraction.

The PAFs of postoperative complications for hospital adverse events, such as reoperation, prolonged hospital stay, and 90-day mortality, were also calculated (*Fig. 3*; *Tables S4–S6*). The complications with the highest contributions to reoperation rates were respiratory failure (PAF 46.0%; 95% c.i. 34.1 to 57.5) and anastomotic leak (PAF 39.8%; 95% c.i. 27.2 to 52.1). Anastomotic leak (PAF 56.9%; 95% c.i. 46.8 to 66.2) had the highest contribution to prolonged hospital stay, whereas the complications with highest contributions to 90-day mortality were respiratory failure (PAF 53.5%; 95% c.i. 30.9 to 73.9) and generalized sepsis (PAF 44.2; 95% c.i. 24.5 to 64.6).

### Long-term quality of life

Information about long-term QoL was available for 105 patients (16.6% of the study population; *Fig. S1*). Overviews of the clinicopathological characteristics and complications for this group are provided in *Tables S7* and *S8*. Among the 58 patients (55.2%) who experienced complications and for whom QoL information was available, pulmonary complications were the most common, occurring in 28% of patients, followed by infectious complications (24%). Eighteen patients (17%) experienced an anastomotic leak. Complications that affected more than 10% of the study population were considered for further analysis.

No complication group or complication type had any effect on long-term HRQoL. After anastomotic leak, patients reported significantly more financial difficulties, with a mean score difference of 17.3 (95% c.i. 6.5 to 28.0; *P* = 0.018), as well as higher amount of insomnia (mean score difference 17.6; 95% c.i. 4.7 to 30.4; *P* = 0.036). Spider plots of the relevant results are shown in *Fig. S2*, with the rest of the findings provided in *Table S9*.

## Discussion

Despite advances in perioperative care and the introduction of multimodal treatment strategies, complications in oesophageal surgery remain common and carry profound consequences. This study assessed the impact of postoperative complications on long-term survival and recurrence, adverse in-hospital outcomes, and QoL in a cohort of 632 patients undergoing oesophageal cancer surgery in a high-volume tertiary centre.

The findings of the present study are consistent with those of previous studies^33,34^ reporting pulmonary, gastrointestinal, and infectious complications as the most prevalent and severe, particularly anastomotic leak and pneumonia. However, in the present study, the traditional survival analysis was extended by integrating adjusted HRs from the Cox regression and PAF to quantify the contribution of specific complications to OS and RFS, offering a novel perspective that has not been widely explored in the current literature and this patient cohort^26,27^.

Pulmonary complications were the most frequent in the present cohort, affecting 31% of patients, with pneumonia specifically contributing significantly to both OS and RFS. This aligns with previous research^35^ highlighting the vulnerability of oesophageal cancer patients to pulmonary complications due to the complexity of thoracoabdominal procedures and the high perioperative risk for respiratory issues. The highest adjusted PAF for pneumonia indicated that a significant proportion of the poor survival outcomes could be prevented if this complication was effectively managed. These results emphasize the need for enhanced, evidence-based perioperative pulmonary care in this patient cohort, such as the implementation of perioperative care bundles^36,37^.

One of the most notable findings of this study was the significant impact of gastrointestinal complications, particularly anastomotic leak, on both reoperation rates and prolonged hospital stays. Anastomotic leak was associated with worse survival outcomes even after 1-year conditional survival analysis. Following pneumonia, anastomotic leak had the highest PAF for both OS and RFS, with estimates ranging from 9.7 to 5.3% after 5 years. This means that preventing anastomotic leaks could potentially reduce the number of deaths or recurrences by up to 9.7% in the first year and by 5.3% after 5 years, highlighting the substantial impact of this complication on long-term outcomes. Although the findings of the present study, along with findings from other studies, underscore the importance of this complication for in-hospital adverse events^10^, survival outcomes^21^, and short- and long-term HRQoL^38^, there is a notable lack of robust evidence for postoperative protocols and from randomized clinical trials on how best to prevent and manage anastomotic leak^39,40^. Moreover, a universally accepted system for documenting the occurrence and severity of complications, as well as quality control measures, is still lacking. However, significant efforts for benchmarking and standardization, such as those from the ECCG, are ongoing, and it is anticipated that stronger evidence will emerge in coming years^5,12^. Prehabilitation measures^41^ and a focus on the early detection of postoperative complications through the identification of ‘index’ complications^4^ could further enhance the management of this vulnerable patient cohort.

The PAF estimates for hospital outcomes in the present study were generally similar with previously published data^10^. In the present study, pulmonary complications and anastomotic leak demonstrated comparable PAFs for 90-day mortality to those reported previously^10^ for 30-day mortality (38.8 *versus* 44.1% and 37.7 *versus* 30.4%, respectively). In the present cohort, respiratory failure and generalized sepsis were additional major contributors to mortality, although these were not evaluated separately in the previous study^10^. Regarding prolonged hospital stay, the PAF for anastomotic leakage was higher in the present cohort than in the previous study^10^ (56.9 *versus* 30.9%), as was the PAF for pulmonary complications (42.3 *versus* 31.4%). For reoperation, PAFs for anastomotic leakage were comparable between the present and previous study^10^ (39.8 *versus* 47.1%), whereas pulmonary complications had a higher impact in the present cohort (38.6 *versus* 17.7%). The differences between studies may be explained by variations in patient characteristics, including a higher proportion of patients with ASA grade II and minimally invasive resections in the Dutch cohort^10^, and a higher burden of severe co-morbidities and open resections in the present study population.

The present study showed that although postoperative complications did not significantly impact long-term HRQoL, specific complications, such as anastomotic leak, were associated with increased financial difficulties and insomnia. However, given the limited availability of QoL data and the assessment of QoL at a single time point, these findings should be interpreted with caution. Moreover, the HRQoL analysis focused specifically on lasting symptoms in patients who survived at least 2 years after surgery; thus, HRQoL data were not available for patients who died within this timeframe, including those contributing to the observed mortality rate. There is, in general, a lack of information about the HRQoL after oesophagectomy in the early postoperative phase, especially after the introduction of multimodal and minimally invasive treatment strategies and outside of clinical trials. Efforts such as the SISAQOL initiative^23^ provide a valuable framework for the standardized and meaningful analysis of HRQoL outcomes in oncological studies, and should be more widely adopted in upper gastrointestinal surgical research and routine clinical practice.

Additional limitations of the study include its single-centre design. The validity and applicability of PAF estimates depend heavily on the prevalence of the exposure (in this case, postoperative complications), which can vary substantially across centres due to differences in surgical volume, technique, perioperative care, and patient selection. The Department of General, Abdominal and Transplantation Surgery at the University Hospital in Heidelberg is a tertiary referral centre in Germany and treats a high proportion of patients with complex co-morbidities and more advanced tumour stages.

Compared with a recent multicentre European cohort^42^, the cohort in the present study had a higher proportion of complications like anastomotic leakage and pneumonia. This can be attributed, in part, to a significantly lower proportion of patients with ASA grade I–II (42 *versus* up to 78.5%), a markedly higher burden of severe co-morbidities, and a lower rate of minimally invasive resections in the present study. Furthermore, a greater proportion of patients in the present study presented with locally advanced tumours (pT3/4 53.2 *versus* 38.8–49.8% in the previous study^42^). All these factors are associated with an increased risk of postoperative complications^43^.Therefore, although the findings of the present study provide valuable insights into the proportional contribution of specific complications in a complex surgical population, caution is warranted when extrapolating the absolute PAF values to centres with different patient populations, surgical approaches, or perioperative strategies.

Furthermore, the low number of patients with certain complications, such as chyle leak, limits the ability to draw definitive conclusions regarding these outcomes.

In conclusion, this study highlights the significant impact of postoperative complications, particularly pneumonia and anastomotic leak, on long-term survival, recurrence, and hospital outcomes in patients with oesophageal and gastro-oesophageal junction cancer. The use of adjusted HRs in combination with PAFs provided a novel perspective on the proportional contribution of these complications to deaths and recurrence at certain time points after surgery. These findings underscore the need for targeted strategies to prevent and manage complications through perioperative care bundles and early detection measures, and emphasize the importance of ongoing research to establish standardized management protocols in this high-risk patient population.

## Supplementary Material

zraf083_Supplementary_Data

## Data Availability

The data sets generated and analysed during the present study are available from the corresponding author upon reasonable request. Due to privacy and ethical considerations, there may be some restrictions regarding data availability.
