# Supplementary material for: Effects of postoperative complications in oesophageal cancer on survival, hospital outcomes, and long-term quality of life: retrospective cohort study
Source: BJS Open. 2025 Aug 2;9(4):zraf083. doi: 10.1093/bjsopen/zraf083 (PMC12317276; doi:10.1093/bjsopen/zraf083)
Supplement: zraf083_Supplementary_Data [file zraf083_supplementary_data.docx]

**The Burden of Postoperative Complications in Esophageal Cancer - Quantifying Their Effect on Survival, Hospital Outcomes, and long-term Quality of Life: Retrospective cohort study**

Nerma Crnovrsanin^1^, Stefan Giring^1^, Antonia Oppel^1^, Ingmar F. Rompen^1^, Sabine Schiefer^1^, Nicolas Jorek^1^, Thomas Schmidt^2^, Beat Müller-Stich^3^, Leila Sisic^1^, Henrik Nienhüser^1^

^1^Department of General, Abdominal and Transplantation Surgery, University Hospital Heidelberg, Heidelberg, Germany

^2^Department of General, Abdominal, Thoracic and Transplantation Surgery, University Hospital Cologne, Cologne, Germany

^3^Department of Surgery, Clarunis University Center for Gastrointestinal and Liver Disease, University Hospital and St. Clare Hospital Basel, Basel, Switzerland.

Corresponding Author:

Henrik Nienhüser, MD

Department of General, Visceral, and Transplantation Surgery, Heidelberg University Hospital

Im Neuenheimer Feld 420, 69120 Heidelberg, Germany

[henrik.nienhueser@med.uni-heidelberg.de](mailto:henrik.nienhueser@med.uni-heidelberg.de)

**Supplementary Materials - Index**

| **Supplementary Figures and Tables** |  |
| --- | --- |
| **Supp. Figure 1**. Flowchart of patient inclusion for the HRQoL analysis. | *Page 3* |
| **Supp. Figure 2.** Spider plot of the significant impact of specific complications on QoL related outcomes. | *Page 4* |
| **Supp. Table 1.** Results of the log-rank test and the probabilities after 5 years for OS and RFS for certain complication groups and specific complications. | *Page 5* |
| **Supp. Table 2.** Clinicopathological characteristics of patients where QoL information was available and hypothesis testing between patients that had any kind of complication and those without. | *Page 5* |
| **Supp. Table 2.** PAF and 95%CI of postoperative complication types and their effect on OS and RFS after 6, 12, 18, 24, 36, 48 and 60 months | *Page 6* |
| **Supp. Table 3.** PAF and 95%CI of postoperative specific complication types and their effect on OS and RFS after 6, 12, 18, 24, 36, 48 and 60 months | *Page 7* |
| **Supp. Table 4.** PAF and 95%CI of postoperative complications and their effect on reoperations. | *Page 9* |
| **Supp. Table 5.** PAF and 95%CI of postoperative complications and their effect on prolonged hospital stay. | *Page 10* |
| **Supp. Table 6.** PAF and 95%CI of postoperative complications and their effect on 90-day mortality. | *Page 11* |
| **Supp. Table 7.** Clinicopathological characteristics of patients where QoL information was available and hypothesis testing between patients that had any kind of complication and those without. | *Page 12* |
| **Supp. Table 8.** Overview of the complications in patients where information about QoL where available. | *Page 14* |
| **Supp. Table 9.** Univariable linear regression of HRQL comparing patients with an without a specific complication | *Page 16* |

**Supplementary Figures and Tables**

**Supp. Figure 1**. Flowchart of patient inclusion for the HRQoL analysis.


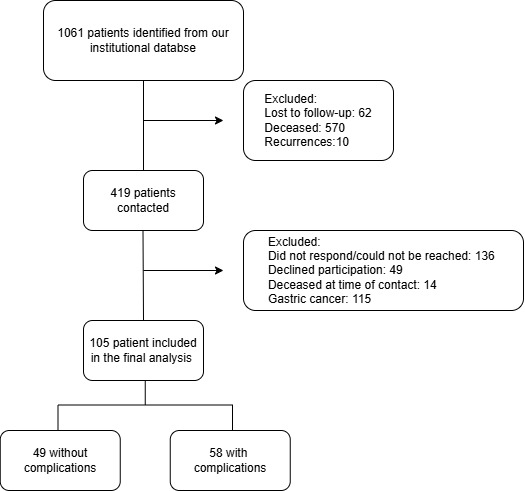


**Supp. Figure 2.** Spider plot of the significant impact of specific complications on QoL-related outcomes. Only values for the symptom scale Insomnia (SL) and Financial difficulties (FI) were significant in the univariate linear regression analysis and had a mean score difference >10 for anastomotic leak. A higher score for a symptom scale represents a high level of symptomatology/problems. FA = Fatigue; NV = Nausea and vomiting; DY = Dyspnea; PA: Pain; AP = Appetite loss; CO = Constipation; DI = Diarrhea.


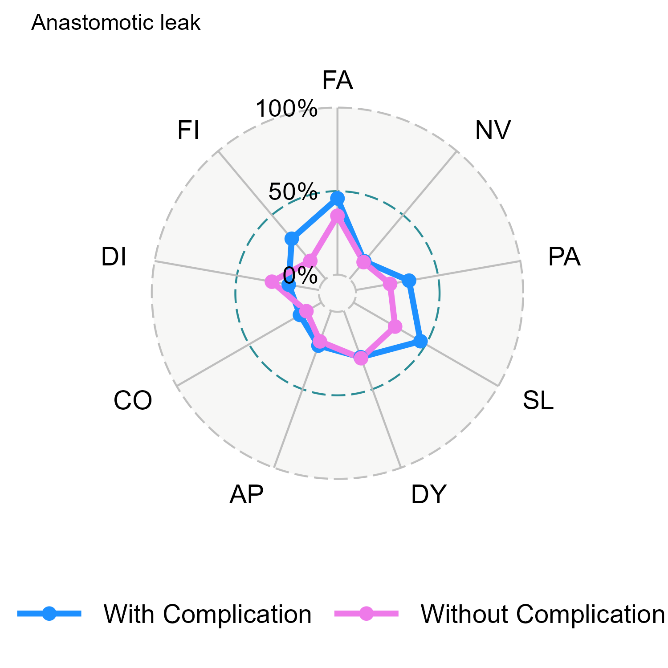


**Supp. Table 1.** Results of the log-rank test and the probabilities after 5 years for OS and RFS for certain complication groups and specific complications.

| Complication | Overall survival | | |  | Recurrence-free survival | | |
| --- | --- | --- | --- | --- | --- | --- | --- |
|  | **without complication** | **with complication** | **p value** |  | **without complication** | **with complication** | **p value** |
| CD > 0 | 0.56 | 0.46 | 0.070 |  | 0.52 | 0.51 | 0.600 |
| CD > IIIa | 0.54 | 0.40 | **0.001** |  | 0.53 | 0.43 | 0.173 |
| Pulmonary complication | 0.54 | 0.40 | **0.009** |  | 0.53 | 0.47 | 0.052 |
| **Pneumonia** | 0.54 | 0.36 | **0.002** |  | 0.53 | 0.43 | **0.009** |
| **Pleural effusion** | 0.52 | 0.24 | 0.130 |  | 0.52 | 0.30 | 0.191 |
| **Pneumothorax** | 0.50 | 0.58 | 0.479 |  | 0.50 | 0.65 | 0.295 |
| **Respiratory failure requiring reintubation** | 0.52 | 0.38 | **0.009** |  | 0.52 | 0.43 | 0.587 |
| Cardiac complication | 0.52 | 0.38 | **0.033** |  | 0.52 | 0.44 | 0.195 |
| **Dysrhythmia requiring treatment** | 0.52 | 0.34 | **0.015** |  | 0.51 | 0.49 | 0.465 |
| Gastrointestinal complication | 0.54 | 0.34 | **0.002** |  | 0.54 | 0.37 | **0.016** |
| **Anastomotic leak** | 0.53 | 0.41 | 0.077 |  | 0.54 | 0.38 | **0.027** |
| **Conduit necrosis** | 0.51 | 0.25 | **0.043** |  | 0.51 | 0.38 | 0.273 |
| **Pylorospasm** | 0.51 | 0.44 | 0.301 |  | 0.51 | 0.63 | 0.263 |
| **Bleeding requiring intervention** | 0.51 | 0.36 | **0.015** |  | 0.52 | 0.19 | 0.168 |
| **Delayed gastric emptying** | 0.51 | 0.38 | 0.463 |  | 0.52 | 0.47 | 0.235 |
| Infectious complication | 0.53 | 0.43 | 0.104 |  | 0.53 | 0.47 | 0.061 |
| **Wound infection** | 0.51 | 0.47 | 0.361 |  | 0.51 | 0.51 | 0.523 |
| **Intrathoracic/intra-abdominal abscess** | 0.53 | 0.33 | **0.010** |  | 0.52 | 0.48 | 0.151 |
| **Generalized sepsis** | 0.51 | 0.30 | **0.041** |  | 0.52 | 0.40 | **0.024** |
| Urological complication | 0.51 | 0.43 | 0.497 |  | 0.51 | 0.52 | 0.816 |
| Thromboembolic complication | 0.51 | 0.48 | 0.747 |  | 0.52 | 0.44 | 0.781 |
| Neurological/psychiatric complication | 0.51 | 0.47 | 0.591 |  | 0.51 | 0.55 | 0.735 |
| Wound/diaphragm complication | 0.51 | 0.50 | 0.532 |  | 0.51 | 0.50 | 0.494 |
| Other complication | 0.51 | 0.49 | 0.604 |  | 0.51 | 0.54 | 0.827 |

**Supp. Table 2.** PAF and 95%CI of postoperative complication types and their effect on OS and RFS after 6, 12, 18, 24, 36, 48 and 60 months adjusted for age, (y)pN, (y)pM, R-Status, ASA classification, perioperative treatment, severe comorbidities, type of surgery and histopathological subtype. Complication types and specific complications that were significant in Table 3 and had more than 10 events were analyzed.

a)

| Complication | Overall survival | |  | Recurrence-free  survival | | Time |
| --- | --- | --- | --- | --- | --- | --- |
|  | **Adj. PAF (95%CI)** | **P - Value** |  | **Adj. PAF (95%CI)** | **P - Value** |  |
| Clavien-Dindo > IIIa | 16.1[5.3-26.9] | 0.004 |  |  |  | 6 |
| Clavien-Dindo > IIIa | 14.2[4.8-23.6] | 0.003 |  |  |  | 12 |
| Clavien-Dindo > IIIa | 13.1[4.4-21.7] | 0.003 |  |  |  | 18 |
| Clavien-Dindo > IIIa | 11.7[4.1-19.4] | 0.003 |  |  |  | 24 |
| Clavien-Dindo > IIIa | 10.1[3.6-16.6] | 0.002 |  |  |  | 36 |
| Clavien-Dindo > IIIa | 8.9[3.2-14.6] | 0.002 |  |  |  | 48 |
| Clavien-Dindo > IIIa | 8.4[3.1-13.8] | 0.002 |  |  |  | 60 |
| Pulmonary | 15.4[4.4-26.4] | 0.006 |  |  |  | 6 |
| Pulmonary | 13.4[3.9-23.0] | 0.006 |  |  |  | 12 |
| Pulmonary | 12.3[3.5-21.1] | 0.006 |  |  |  | 18 |
| Pulmonary | 10.9[3.2-18.7] | 0.006 |  |  |  | 24 |
| Pulmonary | 9.3[2.7-16.0] | 0.006 |  |  |  | 36 |
| Pulmonary | 8.2[2.4-14.0] | 0.006 |  |  |  | 48 |
| Pulmonary | 7.8[2.3-13.2] | 0.005 |  |  |  | 60 |
| Gastrointestinal | 13.7[4.4-22.9] | 0.004 |  | 10.6[2.9-18.3] | 0.007 | 6 |
| Gastrointestinal | 12.1[4.1-20.1] | 0.003 |  | 8.9[2.6-15.1] | 0.006 | 12 |
| Gastrointestinal | 11.1[3.8-18.4] | 0.003 |  | 8.0[2.4-13.6] | 0.005 | 18 |
| Gastrointestinal | 9.9[3.5-16.3] | 0.002 |  | 7.4[2.3-12.5] | 0.005 | 24 |
| Gastrointestinal | 8.5[3.1-13.8] | 0.002 |  | 6.9[2.2-11.7] | 0.004 | 36 |
| Gastrointestinal | 7.5[2.8-12.1] | 0.002 |  | 6.6[2.1-11.1] | 0.004 | 48 |
| Gastrointestinal | 7.1[2.7-11.4] | 0.001 |  | 6.3[2.0-10.5] | 0.004 | 60 |

**Supp. Table 3.** PAF and 95%CI of postoperative specific complication types and their effect on OS and RFS after 6, 12, 18, 24, 36, 48 and 60 months adjusted for age, (y)pN, (y)pM, R-Status, ASA classification, perioperative treatment, severe comorbidities, type of surgery and histopathological subtype. Complication types and specific complications that were significant in Table 3 and had more than 10 events were analyzed. DGE = Delayed gastric emptying.

| Complication | Overall survival | |  | Recurrence-free  survival | | | Time |
| --- | --- | --- | --- | --- | --- | --- | --- |
|  | **Adj. PAF (95%CI)** | **P - Value** |  | **Adj. PAF (95%CI)** | **P - Value** | |  |
| Pneumonia | 12.0[2.4-21.6] | 0.015 |  | 8.1[-0.4-16.5] | | 0.061 | 6 |
| Pneumonia | 10.3[2.1-18.5] | 0.014 |  | 6.6[-0.1-13.3] | | 0.055 | 12 |
| Pneumonia | 9.3[1.9-16.8] | 0.013 |  | 5.9[-0.0-11.8] | | 0.052 | 18 |
| Pneumonia | 8.3[1.8-14.7] | 0.013 |  | 5.4[0.0-10.8] | | 0.049 | 24 |
| Pneumonia | 7.0[1.6-12.5] | 0.012 |  | 5.0[0.1-10.0] | | 0.047 | 36 |
| Pneumonia | 6.2[1.5-10.9] | 0.010 |  | 4.8[0.1-9.5] | | 0.046 | 48 |
| Pneumonia | 5.9[1.4-10.3] | 0.010 |  | 4.5[0.1-8.9] | | 0.044 | 60 |
| Respiratory failure | 8.5[0.7-16.4] | 0.034 |  |  |  | | 6 |
| Respiratory failure | 7.3[0.8-13.7] | 0.028 |  |  |  | | 12 |
| Respiratory failure | 6.6[0.8-12.4] | 0.025 |  |  |  | | 18 |
| Respiratory failure | 5.8[0.9-10.8] | 0.021 |  |  |  | | 24 |
| Respiratory failure | 5.0[0.9-9.1] | 0.017 |  |  |  | | 36 |
| Respiratory failure | 4.4[0.9-7.9] | 0.014 |  |  |  | | 48 |
| Respiratory failure | 4.2[0.9-7.5] | 0.013 |  |  |  | | 60 |
| Anastomotic leak | 9.7[1.5-17.9] | 0.020 |  | 9.4[2.2-16.6] | 0.011 | | 6 |
| Anastomotic leak | 8.7[1.5-15.9] | 0.018 |  | 7.9[2.0-13.9] | 0.009 | | 12 |
| Anastomotic leak | 8.1[1.5-14.6] | 0.016 |  | 7.2[1.9-12.4] | 0.008 | | 18 |
| Anastomotic leak | 7.3[1.5-13.1] | 0.014 |  | 6.6[1.8-11.5] | 0.007 | | 24 |
| Anastomotic leak | 6.3[1.4-11.2] | 0.012 |  | 6.2[1.7-10.7] | 0.007 | | 36 |
| Anastomotic leak | 5.5[1.3-9.8] | 0.011 |  | 5.9[1.6-10.2] | 0.007 | | 48 |
| Anastomotic leak | 5.3[1.2-9.3] | 0.011 |  | 5.6[1.6-9.6] | 0.006 | | 60 |
| DGE | 4.1[-0.8-9.1] | 0.104 |  |  |  | | 6 |
| DGE | 3.9[-0.7-8.5] | 0.093 |  |  |  | | 12 |
| DGE | 3.8[-0.5-8.1] | 0.086 |  |  |  | | 18 |
| DGE | 3.6[-0.4-7.5] | 0.077 |  |  |  | | 24 |
| DGE | 3.2[-0.2-6.6] | 0.065 |  |  |  | | 36 |
| DGE | 2.9[-0.1-5.9] | 0.056 |  |  |  | | 48 |
| DGE | 2.8[-0.0-5.6] | 0.052 |  |  |  | | 60 |
| Generalized sepsis | |  |  | 4.0[-0.7-8.8] | 0.095 | | 6 |
| Generalized sepsis | |  |  | 3.1[-0.2-6.4] | 0.069 | | 12 |
| Generalized sepsis | |  |  | 2.6[-0.1-5.3] | 0.058 | | 18 |
| Generalized sepsis | |  |  | 2.3[-0.0-4.7] | 0.052 | | 24 |
| Generalized sepsis | |  |  | 2.1[0.0-4.2] | 0.048 | | 36 |
| Generalized sepsis | |  |  | 2.0[0.0-3.9] | 0.046 | | 48 |
| Generalized sepsis | |  |  | 1.8[0.0-3.6] | 0.044 | | 60 |

**Supp. Table 4.** PAF and 95%CI of postoperative complications and their effect on reoperations. Adjusted for age, ASA classification, BMI, severe comorbidities, surgical access (open vs. minimally invasive) and type of surgery.

a)

| Complication | No. with/without reoperation | Adjusted RR (95% CI) | p-value | Adjusted PAF (95% CI) | p-value |
| --- | --- | --- | --- | --- | --- |
| Pulmonary complication | 62/45 | 3.00 ( 2.02 - 4.47 ) | <0.001 | 38.6 ( 24.4 - 52.2 ) | <0.001 |
| **Pneumonia** | 48/59 | 2.55 ( 1.70 - 3.79 ) | <0.001 | 27.0 ( 14.3 - 40.0 ) | <0.001 |
| **Pleural effusion** | 22/85 | 1.84 ( 1.12 - 2.92 ) | 0.003 | 9.3 ( 1.4 - 19.0 ) | 0.038 |
| **Pneumothorax** | 15/92 | 2.41 ( 1.34 - 4.06 ) | <0.001 | 8.4 ( 2.1 - 16.6 ) | 0.022 |
| **Respiratory failure requiring reintubation** | 58/49 | 5.89 ( 3.97 - 8.79 ) | <0.001 | 46.0 ( 34.1 - 57.5 ) | <0.001 |
| Cardiac complication | 41/66 | 3.01 ( 2.00 - 4.48 ) | <0.001 | 25.7 ( 14.7 - 37.5 ) | <0.001 |
| **Dysrhythmia requiring treatment** | 22/85 | 2.16 ( 1.29 - 3.47 ) | <0.001 | 11.1 ( 3.1 - 21.0 ) | 0.015 |
| Gastrointestinal complication | 60/47 | 4.12 ( 2.79 - 6.14 ) | <0.001 | 43.0 ( 30.2 - 55.4 ) | <0.001 |
| **Anastomotic leak** | 56/51 | 3.96 ( 2.68 - 5.87 ) | <0.001 | 39.8 ( 27.2 - 52.1 ) | <0.001 |
| **Conduit necrosis** | 13/94 | 5.01 ( 2.62 - 8.85 ) | <0.001 | 10.2 ( 4.4 - 18.3 ) | 0.004 |
| **Pylorospasm** | 6/101 | 1.30 ( 0.50 - 2.75 ) | 0.478 | 1.3 ( -2.2 - 7.2 ) | 0.585 |
| **Bleeding requiring intervention** | 20/87 | 4.84 ( 2.85 - 7.85 ) | <0.001 | 13.6 ( 7.1 - 22.0 ) | <0.001 |
| **Delayed gastric emptying** | 5/102 | 0.53 ( 0.16 - 1.29 ) | 0.196 | -3.5 ( -6.5 - 2.1 ) | 0.107 |
| Infectious complication | 60/47 | 3.02 ( 2.04 - 4.50 ) | <0.001 | 36.8 ( 23.1 - 50.2 ) | <0.001 |
| **Wound infection** | 17/90 | 2.38 ( 1.34 - 3.96 ) | <0.001 | 8.7 ( 2.3 - 17.1 ) | 0.020 |
| **Intrathoracic/intra-abdominal abscess** | 39/68 | 2.98 ( 1.97 - 4.43 ) | <0.001 | 23.3 ( 13.0 - 34.5 ) | <0.001 |
| **Generalized sepsis** | 35/72 | 5.59 ( 3.62 - 8.48 ) | <0.001 | 27.4 ( 17.7 - 38.1 ) | <0.001 |
| Urological complication | 14/93 | 2.13 ( 1.15 - 3.64 ) | 0.001 | 6.7 ( 1.0 - 14.3 ) | 0.050 |
| Thromboembolic complication | 13/94 | 2.22 ( 1.15 - 3.90 ) | 0.001 | 6.1 ( 0.8 - 13.5 ) | 0.058 |
| Neurological/psychiatric complication | 15/92 | 2.12 ( 1.17 - 3.58 ) | 0.001 | 7.5 ( 1.2 - 15.8 ) | 0.043 |
| Wound/diaphragm complication | 24/83 | 3.11 ( 1.88 - 4.97 ) | <0.001 | 14.6 ( 6.6 - 24.2 ) | 0.001 |
| Other complication | 52/55 | 4.52 ( 3.07 - 6.63 ) | <0.001 | 37.1 ( 25.8 - 48.6 ) | <0.001 |

**Supp. Table 5.** PAF and 95%CI of postoperative complications and their effect on prolonged hospital stay. Adjusted for age, ASA classification, BMI, severe comorbidities, surgical access (open vs. minimally invasive) and type of surgery.

| Complication | No. with/without prolonged hospital stay | Adjusted RR (95% CI) | p-value | Adjusted PAF (95% CI) | p-value |
| --- | --- | --- | --- | --- | --- |
| Pulmonary complication | 100/65 | 3.33 ( 2.39 - 4.66 ) | <0.001 | 42.3 ( 30.5 - 53.6 ) | <0.001 |
| **Pneumonia** | 76/89 | 2.53 ( 1.82 - 3.52 ) | <0.001 | 26.8 ( 16.3 - 37.6 ) | <0.001 |
| **Pleural effusion** | 45/120 | 2.54 ( 1.76 - 3.61 ) | <0.001 | 15.8 ( 8.5 - 24.2 ) | <0.001 |
| **Pneumothorax** | 18/147 | 1.84 ( 1.09 - 2.93 ) | 0.001 | 5.2 ( 0.6 - 11.1 ) | 0.056 |
| **Respiratory failure requiring reintubation** | 81/84 | 4.30 ( 3.12 - 5.92 ) | <0.001 | 36.5 ( 27.0 - 46.1 ) | <0.001 |
| Cardiac complication | 61/104 | 2.66 ( 1.89 - 3.72 ) | <0.001 | 22.3 ( 13.3 - 31.9 ) | <0.001 |
| **Dysrhythmia requiring treatment** | 34/131 | 1.87 ( 1.23 - 2.77 ) | <0.001 | 8.6 ( 2.4 - 16.0 ) | 0.014 |
| Gastrointestinal complication | 116/49 | 7.48 ( 5.30 - 10.72 ) | <0.001 | 61.1 ( 51.0 - 70.2 ) | <0.001 |
| **Anastomotic leak** | 110/55 | 6.91 ( 4.95 - 9.76 ) | <0.001 | 56.9 ( 46.8 - 66.2 ) | <0.001 |
| **Conduit necrosis** | 13/152 | 2.70 ( 1.45 - 4.61 ) | <0.001 | 4.6 ( 1.3 - 9.3 ) | 0.025 |
| **Pylorospasm** | 8/157 | 1.29 ( 0.58 - 2.47 ) | 0.394 | 1.3 ( -1.9 - 6.1 ) | 0.541 |
| **Bleeding requiring intervention** | 18/147 | 2.51 ( 1.44 - 4.10 ) | <0.001 | 5.8 ( 1.8 - 11.3 ) | 0.017 |
| **Delayed gastric emptying** | 10/155 | 0.77 ( 0.38 - 1.40 ) | 0.372 | -1.7 ( -4.7 - 2.9 ) | 0.384 |
| Infectious complication | 91/74 | 2.87 ( 2.08 - 3.98 ) | <0.001 | 35.0 ( 23.7 - 46.2 ) | <0.001 |
| **Wound infection** | 13/152 | 1.11 ( 0.59 - 1.90 ) | 0.670 | 0.7 ( -2.9 - 5.9 ) | 0.747 |
| **Intrathoracic/intra-abdominal abscess** | 68/97 | 3.86 ( 2.77 - 5.34 ) | <0.001 | 30.5 ( 21.4 - 40.0 ) | <0.001 |
| **Generalized sepsis** | 42/123 | 3.46 ( 2.36 - 4.96 ) | <0.001 | 16.8 ( 10.1 - 24.6 ) | <0.001 |
| Urological complication | 17/148 | 1.52 ( 0.87 - 2.49 ) | 0.039 | 3.2 ( -0.8 - 8.6 ) | 0.185 |
| Thromboembolic complication | 22/143 | 2.73 ( 1.65 - 4.29 ) | <0.001 | 8.5 ( 3.4 - 15.0 ) | 0.004 |
| Neurological/psychiatric complication | 25/140 | 2.04 ( 1.28 - 3.11 ) | <0.001 | 7.0 ( 2.0 - 13.3 ) | 0.015 |
| Wound/diaphragm complication | 16/149 | 1.19 ( 0.67 - 1.98 ) | 0.404 | 1.5 ( -2.7 - 7.3 ) | 0.548 |
| Other complication | 50/115 | 2.10 ( 1.48 - 2.94 ) | <0.001 | 15.6 ( 7.4 - 24.6 ) | <0.001 |

**Supp. Table 6.** PAF and 95%CI of postoperative complications and their effect on 90-day mortality. Adjusted for age, ASA classification, BMI, severe comorbidities, surgical access (open vs. minimally invasive) and type of surgery.

| Complication | No. with/without 90-day mortailty | Adjusted RR (95% CI) | p-value | | Adjusted PAF (95% CI) | p-value |
| --- | --- | --- | --- | --- | --- | --- |
| Pulmonary complication | 19/12 | 3.01 ( 1.41 - 6.70 ) | | 0.005 | 38.8 ( 11.6 - 64.2 ) | 0.004 |
| **Pneumonia** | 16/15 | 2.62 ( 1.22 - 5.64 ) | | 0.014 | 27.9 ( 5.0 - 52.5 ) | 0.022 |
| **Pleural effusion** | 5/26 | 1.25 ( 0.42 - 3.09 ) | | 0.650 | 3.0 ( -7.7 - 20.3 ) | 0.674 |
| **Pneumothorax** | 2/29 | 0.96 ( 0.15 - 3.20 ) | | 0.953 | -0.3 ( -5.8 - 12.5 ) | 0.950 |
| **Respiratory failure requiring reintubation** | 20/11 | 7.62 ( 3.57 - 17.27 ) | | <0.001 | 53.5 ( 30.9 - 73.9 ) | <0.001 |
| Cardiac complication | 15/16 | 3.66 ( 1.68 - 7.90 ) | | 0.001 | 31.4 ( 10.5 - 54.3 ) | 0.005 |
| **Dysrhythmia requiring treatment** | 8/23 | 2.00 ( 0.76 - 4.69 ) | | 0.116 | 9.7 ( -2.6 - 28.4 ) | 0.219 |
| Gastrointestinal complication | 17/14 | 3.33 ( 1.59 - 7.14 ) | | 0.001 | 36.1 ( 12.4 - 59.8 ) | 0.003 |
| **Anastomotic leak** | 17/14 | 3.71 ( 1.77 - 7.94 ) | | <0.001 | 37.7 ( 14.6 - 60.8 ) | 0.001 |
| **Conduit necrosis** | 7/24 | 9.55 ( 3.75 - 21.53 ) | | <0.001 | 19.6 ( 7.3 - 36.9 ) | 0.010 |
| **Bleeding requiring intervention** | 4/27 | 2.23 ( 0.62 - 6.22 ) | | 0.112 | 4.8 ( -1.6 - 17.7 ) | 0.328 |
| **Delayed gastric emptying** | 1/30 | 0.42 ( 0.02 - 1.99 ) | | 0.377 | -4.4 ( -7.7 - 6.7 ) | 0.230 |
| Infectious complication | 21/10 | 4.53 ( 2.08 - 10.65 ) | | <0.001 | 50.4 ( 23.7 - 73.5 ) | <0.001 |
| **Wound infection** | 5/26 | 1.55 ( 0.44 - 4.25 ) | | 0.357 | 3.7 ( -4.1 - 18.4 ) | 0.520 |
| **Intrathoracic/intra-abdominal abscess** | 6/25 | 1.03 ( 0.37 - 2.44 ) | | 0.944 | 0.5 ( -10.6 - 18.2 ) | 0.947 |
| **Generalized sepsis** | 17/14 | 10.62 ( 4.95 - 23.19 ) | | <0.001 | 44.2 ( 24.5 - 64.6 ) | <0.001 |
| Urological complication | 7/24 | 3.17 ( 1.15 - 7.53 ) | | 0.005 | 12.1 ( 0.9 - 29.3 ) | 0.094 |
| Thromboembolic complication | 5/26 | 2.80 ( 0.82 - 7.30 ) | | 0.046 | 8.8 ( -1.0 - 25.3 ) | 0.188 |
| Neurological/psychiatric complication | 5/26 | 1.66 ( 0.48 - 4.36 ) | | 0.351 | 4.6 ( -3.9 - 19.6 ) | 0.442 |
| Wound/diaphragm complication | 5/26 | 1.29 ( 0.36 - 3.61 ) | | 0.607 | 2.3 ( -5.5 - 17.4 ) | 0.696 |
| Other complication | 12/19 | 2.71 ( 1.22 - 5.76 ) | | 0.010 | 22.3 ( 3.6 - 44.4 ) | 0.032 |

**Supp. Table 7.** Clinicopathological characteristics of patients where QoL information was available and hypothesis testing between patients that had any kind of complication and those without.

| Characteristic | Overall,  N = 105 | No complications,  N = 47 | Complications,  N = 58 | p-value |
| --- | --- | --- | --- | --- |
| Age | 61 (55, 67) | 61 (57, 65) | 62 (54, 67) | 0.834 |
| ASA classification |  |  |  | 0.868 |
| **I/II** | 44 (43%) | 19 (42%) | 25 (44%) |  |
| **III/IV** | 58 (57%) | 26 (58%) | 32 (56%) |  |
| BMI | 26.3 (23.9, 29.4) | 26.9 (23.9, 29.3) | 26.2 (24.6, 29.4) | 0.972 |
| Sex |  |  |  | 0.128 |
| **Female** | 15 (14%) | 4 (8.5%) | 11 (19%) |  |
| **Male** | 90 (86%) | 43 (91%) | 47 (81%) |  |
| Severe comorbidities | 26 (25%) | 13 (28%) | 13 (22%) | 0.494 |
| Cardial comorbidities | 60 (58%) | 28 (61%) | 32 (55%) | 0.559 |
| Pulmonary comorbidities | 17 (16%) | 9 (20%) | 8 (14%) | 0.429 |
| Metabolic comorbidities | 24 (23%) | 9 (20%) | 15 (26%) | 0.449 |
| Histopathologic subtype |  |  |  | 0.536 |
| **Adenocarcinoma** | 94 (90%) | 41 (87%) | 53 (91%) |  |
| **SCC** | 11 (10%) | 6 (13%) | 5 (8.6%) |  |
| cT |  |  |  | 0.735 |
| **cI/II** | 21 (22%) | 10 (24%) | 11 (21%) |  |
| **cIII** | 69 (73%) | 30 (73%) | 39 (74%) |  |
| **cIV** | 4 (4.3%) | 1 (2.4%) | 3 (5.7%) |  |
| cN |  |  |  | 0.349 |
| **cN0** | 22 (21%) | 8 (17%) | 14 (25%) |  |
| **cN1-3** | 82 (79%) | 39 (83%) | 43 (75%) |  |
| cM |  |  |  | 0.729 |
| **cM0** | 97 (92%) | 44 (94%) | 53 (91%) |  |
| **cM1** | 8 (7.6%) | 3 (6.4%) | 5 (8.6%) |  |
| Type of neoadjuvant chemotherapy |  |  |  | 0.733 |
| **none** | 22 (22%) | 12 (26%) | 10 (19%) |  |
| **Epirubicin - based** | 16 (16%) | 6 (13%) | 10 (19%) |  |
| **FLOT/FLO** | 52 (52%) | 23 (50%) | 29 (54%) |  |
| **RCTx** | 10 (10%) | 5 (11%) | 5 (9.3%) |  |
| Interruption of neoadjuvant chemotherapy | 7 (9.0%) | 2 (5.9%) | 5 (11%) | 0.460 |
| pT |  |  |  | 0.963 |
| **p0** | 22 (21%) | 9 (19%) | 13 (22%) |  |
| **pI/II** | 50 (48%) | 23 (49%) | 27 (47%) |  |
| **pIII** | 32 (30%) | 15 (32%) | 17 (29%) |  |
| **pIVa/b** | 1 (1.0%) | 0 (0%) | 1 (1.7%) |  |
| pN |  |  |  | 0.209 |
| **pN0** | 77 (74%) | 32 (68%) | 45 (79%) |  |
| **pN1-3** | 27 (26%) | 15 (32%) | 12 (21%) |  |
| Lymph node ratio | 0.00 (0.00, 0.03) | 0.00 (0.00, 0.04) | 0.00 (0.00, 0.00) | 0.306 |
| pM |  |  |  | >0.999 |
| **pM0** | 104 (99%) | 47 (100%) | 57 (98%) |  |
| **pM1** | 1 (1.0%) | 0 (0%) | 1 (1.7%) |  |
| R status |  |  |  | 0.729 |
| **0** | 97 (92%) | 44 (94%) | 53 (91%) |  |
| **1** | 8 (7.6%) | 3 (6.4%) | 5 (8.6%) |  |
| Complete Pathological Regression | 43 (57%) | 18 (62%) | 25 (53%) | 0.448 |
| Adjuvant treatment | 48 (51%) | 26 (65%) | 22 (41%) | **0.020** |
| Type of surgery |  |  |  | 0.312 |
| **Thoraco-abdominal resection** | 87 (83%) | 37 (79%) | 50 (86%) |  |
| **Transhiatal gastrectomy** | 18 (17%) | 10 (21%) | 8 (14%) |  |
| Localisation of anastomosis |  |  |  | 0.314 |
| **Cervical** | 1 (1.0%) | 0 (0%) | 1 (1.7%) |  |
| **Intraabdominal** | 5 (4.8%) | 4 (8.5%) | 1 (1.7%) |  |
| **Intrahtoracic, level of mediastinum** | 14 (13%) | 6 (13%) | 8 (14%) |  |
| **Intrahtoracic, level of v. azygos** | 85 (81%) | 37 (79%) | 48 (83%) |  |
| Type of reconstruction |  |  |  | 0.464 |
| **Gastric pull-up** | 80 (76%) | 35 (74%) | 45 (78%) |  |
| **Roux-Y** | 18 (17%) | 10 (21%) | 8 (14%) |  |
| **Other** | 7 (6.7%) | 2 (4.3%) | 5 (8.6%) |  |
| Laparoscopic | 18 (17%) | 9 (19%) | 9 (16%) | 0.623 |
| Robotic | 2 (1.9%) | 2 (4.3%) | 0 (0%) | 0.198 |
| Duration of surgery (min) | 280.00 (228.75, 337.00) | 270.00 (225.00, 321.00) | 285.00 (235.00, 340.00) | 0.863 |
| ICU length of stay (days) | 6 (4, 10) | 5 (3, 6) | 8 (6, 20) | **<0.001** |
| Length of stay (days) | 17 (14, 24) | 14 (14, 17) | 23 (17, 34) | **<0.001** |
| Intraoperative complication | 9 (8.6%) | 3 (6.4%) | 6 (10%) | 0.728 |
| Clavien-Dindo classification |  |  |  | **<0.001** |
| **0** | 47 (45%) | 47 (100%) | 0 (0%) |  |
| **I** | 15 (14%) | 0 (0%) | 15 (26%) |  |
| **II** | 9 (8.6%) | 0 (0%) | 9 (16%) |  |
| **IIIa** | 10 (9.5%) | 0 (0%) | 10 (17%) |  |
| **IIIb** | 12 (11%) | 0 (0%) | 12 (21%) |  |
| **IVa** | 10 (9.5%) | 0 (0%) | 10 (17%) |  |
| **IVb** | 2 (1.9%) | 0 (0%) | 2 (3.4%) |  |
| Median (IQR); n (%) | | | | |
| Wilcoxon rank sum test; Pearson's Chi-squared test; Fisher's exact test | | | | |

**Supp. Table 8.** Overview of the complications in patients where information about QoL where available.

| Characteristic | N = 105**^1^** | |
| --- | --- | --- |
| **Patients with Complication** | 58 (55%) | |
| **Clavien-Dindo > IIIa** | 24 (23%) | |
| **Pulmonary complication** | 29 (28%) | |
| Pneumonia | 20 (19%) | |
| Pleural effusion | 6 (5.7%) | |
| Pneumothorax | 5 (4.8%) | |
| Respiratory failure requiring reintubation | 11 (10%) | |
| Tracheobronchial injury | 0 (0%) | |
| **Cardial complication** | 14 (13%) | |
| Cardiac rest requiring CPR | 0 (0%) | |
| Myocardial infarction | 2 (1.9%) | |
| Dysrhythmia requiring treatment | 9 (8.6%) | |
| **Gastrointestinal complication** | 17 (16%) | |
| Anastomotic leak | 18 (17%) | |
| Conduit necrosis | 1 (1.0%) | |
| Ileus | 0 (0%) | |
| Pylorospasm | 3 (2.9%) | |
| Bleeding requiring intervention | 2 (1.9%) | |
| Delayed gastric emptying | 7 (6.7%) | |
| Pancreatitis | 2 (1.9%) | |
| **Infectious complication** | 25 (24%) | |
| Wound infection | 6 (5.7%) | |
| Intrathoracic/intra-abdominal abscess | 13 (12%) | |
| Generalized sepsis | 5 (4.8%) | |
| **Urological complication** | 4 (3.8%) | |
| **Thromboembolic complication** | 9 (8.6%) | |
| **Neurological/psychiatric complication** | 6 (5.7%) | |
| Vocal cord injury/palsy | 1 (1.0%) | |
| **Wound/diaphragm complications** | 8 (7.6%) | |
| Abdominal wall dehiscence/hernia | 3 (2.9%) | |
| Enterothorax | 2 (1.9%) | |
| **Other complication** | 15 (14%) | |
| Chyle leak | 7 (6.7%) | |
| **Conservative management** | 48 (46%) | |
| **Interventional management** | 26 (25%) | |
| **Reoperation** | 12 (11%) | |
| ^1^n (%) | |  |

**Supp. Table 9.** Univariable linear regression of HRQL comparing patients with an without a specific complication

| Complication | Scale | p value | Mean score complication | Mean score without complication | Difference |
| --- | --- | --- | --- | --- | --- |
| Pulmonary | Physical Functioning | 0.582 | 76.6 ( 68.7 - 84.4 ) | 79.0 ( 74.6 - 83.3 ) | -2.4 |
| Pneumonia | Physical Functioning | 0.748 | 77.0 ( 67.7 - 86.3 ) | 78.6 ( 74.4 - 82.8 ) | -1.6 |
| Cardiac | Physical Functioning | 0.995 | 78.3 ( 69.9 - 86.7 ) | 78.3 ( 74.1 - 82.5 ) | 0.0 |
| Gastrointestinal | Physical Functioning | 0.988 | 78.2 ( 68.8 - 87.7 ) | 78.3 ( 74.1 - 82.5 ) | -0.1 |
| Anastomotic leak | Physical Functioning | 0.673 | 76.5 ( 67.9 - 85.1 ) | 78.7 ( 74.4 - 82.9 ) | -2.2 |
| Infectious | Physical Functioning | 0.431 | 81.1 ( 74.1 - 88.0 ) | 77.4 ( 72.9 - 82.0 ) | 3.6 |
| Intrathoracic/intra-abdominal abscess | Physical Functioning | 0.472 | 82.1 ( 70.5 - 93.6 ) | 77.8 ( 73.7 - 81.8 ) | 4.3 |
| Other | Physical Functioning | 0.840 | 77.3 ( 66.9 - 87.8 ) | 78.5 ( 74.3 - 82.6 ) | -1.1 |
| Pulmonary | Role Functioning | 0.423 | 70.7 ( 60.9 - 80.5 ) | 65.8 ( 59.4 - 72.1 ) | 4.9 |
| Pneumonia | Role Functioning | 0.513 | 70.8 ( 59.5 - 82.2 ) | 66.3 ( 60.2 - 72.3 ) | 4.6 |
| Cardiac | Role Functioning | 0.811 | 65.5 ( 52.5 - 78.5 ) | 67.4 ( 61.6 - 73.2 ) | -1.9 |
| Gastrointestinal | Role Functioning | 0.812 | 68.6 ( 56.8 - 80.5 ) | 66.9 ( 60.9 - 72.8 ) | 1.8 |
| Anastomotic leak | Role Functioning | 0.699 | 64.8 ( 53.6 - 76.0 ) | 67.6 ( 61.6 - 73.7 ) | -2.8 |
| Infectious | Role Functioning | 0.123 | 74.7 ( 65.6 - 83.7 ) | 64.8 ( 58.5 - 71.1 ) | 9.9 |
| Intrathoracic/intra-abdominal abscess | Role Functioning | 0.644 | 70.5 ( 58.2 - 82.9 ) | 66.7 ( 60.8 - 72.5 ) | 3.8 |
| Other | Role Functioning | 0.355 | 73.3 ( 56.5 - 90.1 ) | 66.1 ( 60.5 - 71.7 ) | 7.2 |
| Pulmonary | Social Functioning | 0.810 | 67.2 ( 55.6 - 78.9 ) | 68.9 ( 62.0 - 75.7 ) | -1.6 |
| Pneumonia | Social Functioning | 0.600 | 71.7 ( 58.0 - 85.3 ) | 67.6 ( 61.1 - 74.2 ) | 4.0 |
| Cardiac | Social Functioning | 0.942 | 67.9 ( 48.6 - 87.1 ) | 68.5 ( 62.4 - 74.6 ) | -0.6 |
| Gastrointestinal | Social Functioning | 0.588 | 64.7 ( 47.0 - 82.4 ) | 69.1 ( 63.0 - 75.3 ) | -4.4 |
| Anastomotic leak | Social Functioning | 0.335 | 62.0 ( 45.8 - 78.3 ) | 69.7 ( 63.5 - 76.0 ) | -7.7 |
| Infectious | Social Functioning | 0.505 | 72.0 ( 60.1 - 83.9 ) | 67.3 ( 60.5 - 74.1 ) | 4.7 |
| Intrathoracic/intra-abdominal abscess | Social Functioning | 0.706 | 65.4 ( 46.7 - 84.0 ) | 68.8 ( 62.7 - 75.0 ) | -3.5 |
| Other | Social Functioning | 0.715 | 71.1 ( 53.5 - 88.7 ) | 68.0 ( 61.7 - 74.2 ) | 3.1 |
| Pulmonary | Emotional Functioning | 0.637 | 70.1 ( 61.5 - 78.7 ) | 72.5 ( 67.4 - 77.6 ) | -2.4 |
| Pneumonia | Emotional Functioning | 0.226 | 66.3 ( 54.7 - 77.8 ) | 73.1 ( 68.5 - 77.8 ) | -6.9 |
| Cardiac | Emotional Functioning | 0.702 | 69.6 ( 56.1 - 83.2 ) | 72.2 ( 67.6 - 76.8 ) | -2.5 |
| Gastrointestinal | Emotional Functioning | 0.469 | 68.1 ( 55.9 - 80.4 ) | 72.5 ( 67.9 - 77.2 ) | -4.4 |
| Anastomotic leak | Emotional Functioning | 0.340 | 67.1 ( 55.7 - 78.6 ) | 72.8 ( 68.1 - 77.5 ) | -5.7 |
| Infectious | Emotional Functioning | 0.965 | 72.0 ( 63.0 - 81.0 ) | 71.8 ( 66.8 - 76.8 ) | 0.2 |
| Intrathoracic/intra-abdominal abscess | Emotional Functioning | 0.335 | 77.6 ( 64.6 - 90.6 ) | 71.0 ( 66.4 - 75.6 ) | 6.5 |
| Other | Emotional Functioning | 0.234 | 78.3 ( 69.2 - 87.5 ) | 70.7 ( 65.9 - 75.6 ) | 7.6 |
| Pulmonary | Cognitive Functioning | 0.693 | 82.2 ( 73.3 - 91.1 ) | 80.3 ( 75.5 - 85.1 ) | 1.9 |
| Pneumonia | Cognitive Functioning | 0.993 | 80.8 ( 69.4 - 92.3 ) | 80.8 ( 76.3 - 85.3 ) | 0.0 |
| Cardiac | Cognitive Functioning | 0.089 | 71.4 ( 55.2 - 87.6 ) | 82.2 ( 78.1 - 86.4 ) | -10.8 |
| Gastrointestinal | Cognitive Functioning | 0.780 | 79.4 ( 66.4 - 92.4 ) | 81.1 ( 76.6 - 85.5 ) | -1.6 |
| Anastomotic leak | Cognitive Functioning | 0.528 | 77.8 ( 65.7 - 89.9 ) | 81.4 ( 76.9 - 85.9 ) | -3.6 |
| Infectious | Cognitive Functioning | 0.630 | 82.7 ( 73.3 - 92.0 ) | 80.2 ( 75.4 - 85.0 ) | 2.5 |
| Intrathoracic/intra-abdominal abscess | Cognitive Functioning | 0.828 | 82.1 ( 68.5 - 95.6 ) | 80.6 ( 76.1 - 85.1 ) | 1.4 |
| Other | Cognitive Functioning | 0.789 | 82.2 ( 69.3 - 95.2 ) | 80.6 ( 76.1 - 85.0 ) | 1.7 |
| Pulmonary | Fatigue | 0.556 | 39.5 ( 29.5 - 49.5 ) | 36.1 ( 30.4 - 41.8 ) | 3.4 |
| Pneumonia | Fatigue | 0.573 | 40.0 ( 27.0 - 53.0 ) | 36.3 ( 31.0 - 41.7 ) | 3.7 |
| Cardiac | Fatigue | 0.839 | 35.7 ( 19.9 - 51.6 ) | 37.2 ( 32.0 - 42.5 ) | -1.5 |
| Gastrointestinal | Fatigue | 0.292 | 43.1 ( 29.7 - 56.6 ) | 35.9 ( 30.5 - 41.2 ) | 7.3 |
| Anastomotic leak | Fatigue | 0.121 | 45.7 ( 33.5 - 57.9 ) | 35.2 ( 29.9 - 40.6 ) | 10.4 |
| Infectious | Fatigue | 0.795 | 38.2 ( 27.1 - 49.3 ) | 36.7 ( 31.1 - 42.2 ) | 1.6 |
| Intrathoracic/intra-abdominal abscess | Fatigue | 0.424 | 31.6 ( 17.1 - 46.2 ) | 37.8 ( 32.5 - 43.1 ) | -6.2 |
| Other | Fatigue | 0.813 | 35.6 ( 21.9 - 49.2 ) | 37.3 ( 31.9 - 42.6 ) | -1.7 |
| Pulmonary | Pain | 0.356 | 27.0 ( 16.7 - 37.3 ) | 21.3 ( 14.9 - 27.6 ) | 5.7 |
| Pneumonia | Pain | 0.709 | 25.0 ( 12.3 - 37.7 ) | 22.4 ( 16.3 - 28.4 ) | 2.6 |
| Cardiac | Pain | 0.068 | 35.7 ( 18.0 - 53.5 ) | 20.9 ( 15.3 - 26.4 ) | 14.8 |
| Gastrointestinal | Pain | 0.096 | 33.3 ( 15.6 - 51.0 ) | 20.8 ( 15.4 - 26.3 ) | 12.5 |
| Anastomotic leak | Pain | 0.117 | 32.4 ( 15.6 - 49.2 ) | 20.9 ( 15.4 - 26.4 ) | 11.5 |
| Infectious | Pain | 0.566 | 20.0 ( 9.8 - 30.2 ) | 23.8 ( 17.4 - 30.1 ) | -3.8 |
| Intrathoracic/intra-abdominal abscess | Pain | 0.707 | 25.6 ( 6.6 - 44.7 ) | 22.5 ( 16.9 - 28.1 ) | 3.2 |
| Other | Pain | 0.675 | 20.0 ( 7.6 - 32.4 ) | 23.3 ( 17.3 - 29.3 ) | -3.3 |
| Pulmonary | Nausea and Vomiting | 0.601 | 11.5 ( 3.1 - 19.9 ) | 14.0 ( 9.1 - 18.9 ) | -2.5 |
| Pneumonia | Nausea and Vomiting | 0.852 | 14.2 ( 2.5 - 25.8 ) | 13.1 ( 8.7 - 17.6 ) | 1.0 |
| Cardiac | Nausea and Vomiting | 0.699 | 15.5 ( 4.9 - 26.0 ) | 13.0 ( 8.4 - 17.6 ) | 2.5 |
| Gastrointestinal | Nausea and Vomiting | 0.905 | 12.7 ( 3.2 - 22.3 ) | 13.4 ( 8.7 - 18.2 ) | -0.7 |
| Anastomotic leak | Nausea and Vomiting | 0.907 | 13.9 ( 4.6 - 23.1 ) | 13.2 ( 8.5 - 18.0 ) | 0.7 |
| Infectious | Nausea and Vomiting | 0.492 | 10.7 ( 1.1 - 20.3 ) | 14.2 ( 9.5 - 18.9 ) | -3.5 |
| Intrathoracic/intra-abdominal abscess | Nausea and Vomiting | 0.154 | 5.1 ( -4.9 - 15.2 ) | 14.5 ( 9.9 - 19.1 ) | -9.4 |
| Other | Nausea and Vomiting | 0.531 | 10.0 ( 0.0 - 20.0 ) | 13.9 ( 9.2 - 18.5 ) | -3.9 |
| Pulmonary | Sleep Disturbances | 0.719 | 29.9 ( 16.0 - 43.8 ) | 32.5 ( 25.6 - 39.3 ) | -2.6 |
| Pneumonia | Sleep Disturbances | 0.990 | 31.7 ( 14.3 - 49.1 ) | 31.8 ( 25.2 - 38.3 ) | -0.1 |
| Cardiac | Sleep Disturbances | 0.282 | 40.5 ( 22.1 - 58.8 ) | 30.4 ( 23.8 - 37.0 ) | 10.1 |
| Gastrointestinal | Sleep Disturbances | 0.064 | 45.1 ( 24.9 - 65.3 ) | 29.2 ( 22.9 - 35.4 ) | 15.9 |
| Anastomotic leak | Sleep Disturbances | 0.036 | 46.3 ( 27.2 - 65.4 ) | 28.7 ( 22.5 - 35.0 ) | 17.6 |
| Infectious | Sleep Disturbances | 0.850 | 30.7 ( 16.6 - 44.7 ) | 32.1 ( 25.2 - 39.0 ) | -1.4 |
| Intrathoracic/intra-abdominal abscess | Sleep Disturbances | 0.625 | 35.9 ( 14.4 - 57.4 ) | 31.2 ( 24.7 - 37.6 ) | 4.7 |
| Other | Sleep Disturbances | 0.839 | 33.3 ( 15.3 - 51.4 ) | 31.5 ( 24.8 - 38.1 ) | 1.9 |
| Pulmonary | Dyspnea | 0.863 | 31.0 ( 18.5 - 43.6 ) | 29.8 ( 22.8 - 36.8 ) | 1.2 |
| Pneumonia | Dyspnea | 0.424 | 25.0 ( 9.4 - 40.6 ) | 31.4 ( 24.8 - 38.0 ) | -6.4 |
| Cardiac | Dyspnea | 0.426 | 23.8 ( 9.4 - 38.2 ) | 31.1 ( 24.5 - 37.8 ) | -7.3 |
| Gastrointestinal | Dyspnea | 0.226 | 21.6 ( 7.9 - 35.2 ) | 31.8 ( 25.1 - 38.6 ) | -10.2 |
| Anastomotic leak | Dyspnea | 0.939 | 29.6 ( 13.9 - 45.4 ) | 30.3 ( 23.6 - 36.9 ) | -0.6 |
| Infectious | Dyspnea | 0.179 | 22.7 ( 10.9 - 34.4 ) | 32.5 ( 25.4 - 39.6 ) | -9.8 |
| Intrathoracic/intra-abdominal abscess | Dyspnea | 0.246 | 20.5 ( 4.8 - 36.3 ) | 31.5 ( 24.9 - 38.1 ) | -11.0 |
| Other | Dyspnea | 0.456 | 24.4 ( 9.5 - 39.4 ) | 31.1 ( 24.4 - 37.8 ) | -6.7 |
| Pulmonary | Appetite Loss | 0.533 | 23.0 ( 11.7 - 34.3 ) | 18.9 ( 12.1 - 25.6 ) | 4.1 |
| Pneumonia | Appetite Loss | 0.785 | 18.3 ( 5.4 - 31.3 ) | 20.4 ( 13.9 - 26.9 ) | -2.1 |
| Cardiac | Appetite Loss | 0.412 | 26.2 ( 7.8 - 44.5 ) | 19.0 ( 13.0 - 25.1 ) | 7.1 |
| Gastrointestinal | Appetite Loss | 0.601 | 23.5 ( 9.0 - 38.1 ) | 19.3 ( 13.0 - 25.6 ) | 4.2 |
| Anastomotic leak | Appetite Loss | 0.733 | 22.2 ( 8.2 - 36.2 ) | 19.5 ( 13.2 - 25.9 ) | 2.7 |
| Infectious | Appetite Loss | 1.000 | 20.0 ( 7.5 - 32.5 ) | 20.0 ( 13.5 - 26.5 ) | 0.0 |
| Intrathoracic/intra-abdominal abscess | Appetite Loss | 0.558 | 15.4 ( 3.4 - 27.3 ) | 20.7 ( 14.3 - 27.0 ) | -5.3 |
| Other | Appetite Loss | 0.760 | 22.2 ( 5.8 - 38.7 ) | 19.6 ( 13.4 - 25.8 ) | 2.6 |
| Pulmonary | Constipation | 0.471 | 13.8 ( 4.9 - 22.7 ) | 10.1 ( 4.9 - 15.3 ) | 3.7 |
| Pneumonia | Constipation | 0.639 | 13.3 ( 2.3 - 24.3 ) | 10.6 ( 5.7 - 15.5 ) | 2.7 |
| Cardiac | Constipation | 0.588 | 14.3 ( -0.6 - 29.2 ) | 10.6 ( 6.0 - 15.3 ) | 3.7 |
| Gastrointestinal | Constipation | 0.381 | 15.7 ( 3.0 - 28.4 ) | 10.2 ( 5.5 - 15.0 ) | 5.5 |
| Anastomotic leak | Constipation | 0.464 | 14.8 ( 2.7 - 26.9 ) | 10.3 ( 5.5 - 15.2 ) | 4.5 |
| Infectious | Constipation | 0.666 | 9.3 ( 1.3 - 17.4 ) | 11.7 ( 6.3 - 17.0 ) | -2.3 |
| Intrathoracic/intra-abdominal abscess | Constipation | 0.263 | 17.9 ( 3.9 - 32.0 ) | 10.1 ( 5.4 - 14.9 ) | 7.8 |
| Other | Constipation | 0.430 | 15.6 ( 3.0 - 28.1 ) | 10.4 ( 5.6 - 15.2 ) | 5.2 |
| Pulmonary | Diarrhea | 0.560 | 24.1 ( 13.4 - 34.8 ) | 28.1 ( 21.0 - 35.1 ) | -3.9 |
| Pneumonia | Diarrhea | 0.392 | 21.7 ( 9.8 - 33.5 ) | 28.2 ( 21.5 - 34.9 ) | -6.6 |
| Cardiac | Diarrhea | 0.680 | 23.8 ( 11.1 - 36.5 ) | 27.5 ( 21.0 - 34.0 ) | -3.7 |
| Gastrointestinal | Diarrhea | 0.430 | 21.6 ( 9.1 - 34.0 ) | 28.0 ( 21.4 - 34.6 ) | -6.5 |
| Anastomotic leak | Diarrhea | 0.200 | 18.5 ( 7.7 - 29.4 ) | 28.7 ( 22.1 - 35.4 ) | -10.2 |
| Infectious | Diarrhea | 0.121 | 18.7 ( 10.2 - 27.2 ) | 29.6 ( 22.4 - 36.7 ) | -10.9 |
| Intrathoracic/intra-abdominal abscess | Diarrhea | 0.626 | 23.1 ( 9.5 - 36.7 ) | 27.5 ( 21.1 - 34.0 ) | -4.5 |
| Other | Diarrhea | 0.211 | 17.8 ( 9.1 - 26.5 ) | 28.5 ( 21.9 - 35.2 ) | -10.7 |
| Pulmonary | Financial difficulties | 0.597 | 19.5 ( 8.1 - 31.0 ) | 16.2 ( 10.0 - 22.4 ) | 3.3 |
| Pneumonia | Financial difficulties | 0.711 | 15.0 ( 2.0 - 28.0 ) | 17.6 ( 11.6 - 23.7 ) | -2.6 |
| Cardiac | Financial difficulties | 0.350 | 23.8 ( 3.9 - 43.7 ) | 16.1 ( 10.6 - 21.6 ) | 7.7 |
| Gastrointestinal | Financial difficulties | 0.052 | 29.4 ( 11.8 - 47.0 ) | 14.8 ( 9.3 - 20.2 ) | 14.6 |
| Anastomotic leak | Financial difficulties | 0.018 | 31.5 ( 15.2 - 47.7 ) | 14.2 ( 8.7 - 19.7 ) | 17.3 |
| Infectious | Financial difficulties | 0.569 | 20.0 ( 7.5 - 32.5 ) | 16.2 ( 10.2 - 22.3 ) | 3.8 |
| Intrathoracic/intra-abdominal abscess | Financial difficulties | 0.651 | 20.5 ( 3.1 - 37.9 ) | 16.7 ( 10.9 - 22.4 ) | 3.8 |
| Other | Financial difficulties | 0.677 | 20.0 ( 1.1 - 38.9 ) | 16.7 ( 11.1 - 22.3 ) | 3.3 |
| Pulmonary | Quality of Life (Total Score) | 0.867 | 74.4 ( 67.5 - 81.2 ) | 75.0 ( 71.0 - 79.1 ) | -0.7 |
| Pneumonia | Quality of Life (Total Score) | 0.930 | 75.2 ( 66.4 - 84.0 ) | 74.8 ( 71.0 - 78.5 ) | 0.4 |
| Cardiac | Quality of Life (Total Score) | 0.541 | 72.1 ( 61.0 - 83.2 ) | 75.3 ( 71.7 - 78.9 ) | -3.2 |
| Gastrointestinal | Quality of Life (Total Score) | 0.557 | 72.5 ( 62.9 - 82.1 ) | 75.3 ( 71.6 - 79.0 ) | -2.8 |
| Anastomotic leak | Quality of Life (Total Score) | 0.339 | 71.1 ( 62.4 - 79.9 ) | 75.6 ( 71.9 - 79.4 ) | -4.5 |
| Infectious | Quality of Life (Total Score) | 0.345 | 77.9 ( 70.9 - 84.9 ) | 73.9 ( 70.0 - 77.9 ) | 3.9 |
| Intrathoracic/intra-abdominal abscess | Quality of Life (Total Score) | 0.635 | 77.1 ( 66.3 - 87.9 ) | 74.5 ( 70.9 - 78.2 ) | 2.6 |
| Other | Quality of Life (Total Score) | 0.593 | 77.2 ( 67.0 - 87.4 ) | 74.5 ( 70.8 - 78.2 ) | 2.7 |
| Pulmonary | Dysphagia | 0.550 | 11.5 ( 4.0 - 18.9 ) | 14.0 ( 9.8 - 18.3 ) | -2.5 |
| Pneumonia | Dysphagia | 0.887 | 12.8 ( 2.5 - 23.1 ) | 13.5 ( 9.6 - 17.4 ) | -0.7 |
| Cardiac | Dysphagia | 0.237 | 19.0 ( 4.9 - 33.2 ) | 12.5 ( 8.8 - 16.1 ) | 6.6 |
| Gastrointestinal | Dysphagia | 0.586 | 15.7 ( 3.6 - 27.8 ) | 12.9 ( 9.1 - 16.7 ) | 2.8 |
| Anastomotic leak | Dysphagia | 0.615 | 15.4 ( 4.0 - 26.9 ) | 12.9 ( 9.1 - 16.7 ) | 2.5 |
| Infectious | Dysphagia | 0.513 | 11.1 ( 2.6 - 19.6 ) | 14.0 ( 10.0 - 18.1 ) | -2.9 |
| Intrathoracic/intra-abdominal abscess | Dysphagia | 0.542 | 10.3 ( -1.4 - 21.9 ) | 13.8 ( 9.9 - 17.7 ) | -3.5 |
| Other | Dysphagia | 0.524 | 16.3 ( 1.9 - 30.7 ) | 12.8 ( 9.2 - 16.5 ) | 3.5 |
| Pulmonary | Eating | 0.444 | 27.3 ( 18.3 - 36.3 ) | 31.7 ( 25.7 - 37.6 ) | -4.4 |
| Pneumonia | Eating | 0.687 | 28.3 ( 16.8 - 39.8 ) | 30.9 ( 25.4 - 36.5 ) | -2.6 |
| Cardiac | Eating | 0.366 | 36.3 ( 21.0 - 51.6 ) | 29.5 ( 24.3 - 34.8 ) | 6.8 |
| Gastrointestinal | Eating | 0.492 | 26.5 ( 13.1 - 39.8 ) | 31.2 ( 25.9 - 36.6 ) | -4.7 |
| Anastomotic leak | Eating | 0.884 | 29.6 ( 17.2 - 42.0 ) | 30.6 ( 25.2 - 36.1 ) | -1.0 |
| Infectious | Eating | 0.328 | 26.0 ( 17.2 - 34.8 ) | 31.8 ( 25.9 - 37.7 ) | -5.8 |
| Intrathoracic/intra-abdominal abscess | Eating | 0.320 | 23.7 ( 8.9 - 38.5 ) | 31.4 ( 26.1 - 36.7 ) | -7.7 |
| Other | Eating | 0.803 | 28.9 ( 13.4 - 44.4 ) | 30.7 ( 25.5 - 35.9 ) | -1.8 |
| Pulmonary | Reflux | 0.618 | 44.8 ( 33.4 - 56.3 ) | 41.2 ( 33.7 - 48.8 ) | 3.6 |
| Pneumonia | Reflux | 0.587 | 45.8 ( 31.1 - 60.6 ) | 41.4 ( 34.4 - 48.3 ) | 4.5 |
| Cardiac | Reflux | 0.512 | 47.6 ( 30.9 - 64.3 ) | 41.4 ( 34.6 - 48.2 ) | 6.2 |
| Gastrointestinal | Reflux | 0.428 | 48.0 ( 31.5 - 64.6 ) | 41.1 ( 34.3 - 47.9 ) | 6.9 |
| Anastomotic leak | Reflux | 0.272 | 50.0 ( 34.4 - 65.6 ) | 40.6 ( 33.8 - 47.5 ) | 9.4 |
| Infectious | Reflux | 0.700 | 40.0 ( 27.5 - 52.5 ) | 42.9 ( 35.6 - 50.2 ) | -2.9 |
| Intrathoracic/intra-abdominal abscess | Reflux | 0.773 | 39.7 ( 19.6 - 59.8 ) | 42.6 ( 35.9 - 49.2 ) | -2.8 |
| Other | Reflux | 0.482 | 36.7 ( 17.2 - 56.1 ) | 43.1 ( 36.5 - 49.8 ) | -6.5 |
| Pulmonary | Odynophagia | 0.116 | 9.8 ( 3.2 - 16.4 ) | 17.8 ( 12.2 - 23.3 ) | -8.0 |
| Pneumonia | Odynophagia | 0.237 | 10.0 ( 0.7 - 19.3 ) | 16.9 ( 11.8 - 21.9 ) | -6.9 |
| Cardiac | Odynophagia | 0.693 | 17.9 ( 4.4 - 31.3 ) | 15.2 ( 10.5 - 19.9 ) | 2.7 |
| Gastrointestinal | Odynophagia | 0.725 | 13.7 ( 3.2 - 24.3 ) | 15.9 ( 11.0 - 20.8 ) | -2.2 |
| Anastomotic leak | Odynophagia | 0.606 | 13.0 ( 2.9 - 23.0 ) | 16.1 ( 11.1 - 21.1 ) | -3.1 |
| Infectious | Odynophagia | 0.172 | 10.0 ( 2.2 - 17.8 ) | 17.3 ( 12.0 - 22.6 ) | -7.3 |
| Intrathoracic/intra-abdominal abscess | Odynophagia | 0.383 | 10.3 ( -0.6 - 21.1 ) | 16.3 ( 11.5 - 21.1 ) | -6.0 |
| Other | Odynophagia | 1.000 | 15.6 ( -0.2 - 31.3 ) | 15.6 ( 11.0 - 20.1 ) | 0.0 |
| Pulmonary | Pain and discomfort | 0.939 | 24.1 ( 13.3 - 35.0 ) | 23.7 ( 17.9 - 29.5 ) | 0.5 |
| Pneumonia | Pain and discomfort | 0.948 | 24.2 ( 11.1 - 37.2 ) | 23.7 ( 18.1 - 29.3 ) | 0.4 |
| Cardiac | Pain and discomfort | 0.478 | 28.6 ( 12.7 - 44.4 ) | 23.1 ( 17.7 - 28.5 ) | 5.5 |
| Gastrointestinal | Pain and discomfort | 0.907 | 24.5 ( 8.9 - 40.1 ) | 23.7 ( 18.3 - 29.1 ) | 0.8 |
| Anastomotic leak | Pain and discomfort | 0.493 | 27.8 ( 12.6 - 42.9 ) | 23.0 ( 17.6 - 28.4 ) | 4.8 |
| Infectious | Pain and discomfort | 0.419 | 20.0 ( 9.0 - 31.0 ) | 25.0 ( 19.2 - 30.8 ) | -5.0 |
| Intrathoracic/intra-abdominal abscess | Pain and discomfort | 0.774 | 21.8 ( 2.8 - 40.8 ) | 24.1 ( 18.8 - 29.4 ) | -2.3 |
| Other | Pain and discomfort | 0.555 | 20.0 ( 3.7 - 36.3 ) | 24.4 ( 19.1 - 29.8 ) | -4.4 |
| Pulmonary | Anxiety | 0.981 | 43.7 ( 31.3 - 56.1 ) | 43.9 ( 36.1 - 51.6 ) | -0.2 |
| Pneumonia | Anxiety | 0.770 | 45.8 ( 30.5 - 61.2 ) | 43.3 ( 36.1 - 50.6 ) | 2.5 |
| Cardiac | Anxiety | 0.252 | 53.6 ( 33.6 - 73.6 ) | 42.3 ( 35.4 - 49.2 ) | 11.3 |
| Gastrointestinal | Anxiety | 0.730 | 41.2 ( 24.1 - 58.2 ) | 44.3 ( 37.2 - 51.4 ) | -3.1 |
| Anastomotic leak | Anxiety | 0.771 | 41.7 ( 24.9 - 58.4 ) | 44.3 ( 37.1 - 51.4 ) | -2.6 |
| Infectious | Anxiety | 0.849 | 42.7 ( 30.1 - 55.2 ) | 44.2 ( 36.5 - 51.8 ) | -1.5 |
| Intrathoracic/intra-abdominal abscess | Anxiety | 0.980 | 43.6 ( 26.0 - 61.1 ) | 43.8 ( 36.8 - 50.9 ) | -0.3 |
| Other | Anxiety | 0.743 | 41.1 ( 28.4 - 53.8 ) | 44.3 ( 36.9 - 51.6 ) | -3.1 |
| Pulmonary | Eating with others | 0.675 | 10.3 ( 2.3 - 18.4 ) | 12.7 ( 6.6 - 18.8 ) | -2.4 |
| Pneumonia | Eating with others | 0.693 | 10.0 ( -0.7 - 20.7 ) | 12.5 ( 7.0 - 18.1 ) | -2.5 |
| Cardiac | Eating with others | 0.694 | 9.5 ( -4.9 - 23.9 ) | 12.5 ( 7.2 - 17.7 ) | -2.9 |
| Gastrointestinal | Eating with others | 0.695 | 9.8 ( -2.4 - 22.0 ) | 12.5 ( 7.1 - 17.9 ) | -2.7 |
| Anastomotic leak | Eating with others | 0.615 | 9.3 ( -2.3 - 20.8 ) | 12.6 ( 7.2 - 18.1 ) | -3.4 |
| Infectious | Eating with others | 0.136 | 5.3 ( -2.8 - 13.5 ) | 14.2 ( 8.3 - 20.1 ) | -8.8 |
| Intrathoracic/intra-abdominal abscess | Eating with others | 0.789 | 10.3 ( -5.2 - 25.7 ) | 12.3 ( 7.1 - 17.5 ) | -2.1 |
| Other | Eating with others | 0.838 | 13.3 ( -2.0 - 28.7 ) | 11.9 ( 6.7 - 17.0 ) | 1.5 |
| Pulmonary | Dry Mouth | 0.057 | 14.9 ( 6.6 - 23.3 ) | 26.3 ( 19.9 - 32.7 ) | -11.4 |
| Pneumonia | Dry Mouth | 0.139 | 15.0 ( 3.9 - 26.1 ) | 25.1 ( 19.2 - 31.0 ) | -10.1 |
| Cardiac | Dry Mouth | 0.066 | 35.7 ( 17.0 - 54.4 ) | 21.2 ( 16.0 - 26.5 ) | 14.5 |
| Gastrointestinal | Dry Mouth | 0.954 | 23.5 ( 11.3 - 35.8 ) | 23.1 ( 17.3 - 28.9 ) | 0.4 |
| Anastomotic leak | Dry Mouth | 0.872 | 22.2 ( 10.4 - 34.0 ) | 23.4 ( 17.5 - 29.2 ) | -1.1 |
| Infectious | Dry Mouth | 0.348 | 18.7 ( 9.4 - 28.0 ) | 24.6 ( 18.4 - 30.8 ) | -5.9 |
| Intrathoracic/intra-abdominal abscess | Dry Mouth | 0.275 | 15.4 ( 1.3 - 29.5 ) | 24.3 ( 18.6 - 29.9 ) | -8.9 |
| Other | Dry Mouth | 0.413 | 17.8 ( 7.0 - 28.6 ) | 24.1 ( 18.2 - 29.9 ) | -6.3 |
| Pulmonary | Trouble with taste | 0.903 | 16.1 ( 5.5 - 26.7 ) | 15.4 ( 9.2 - 21.5 ) | 0.7 |
| Pneumonia | Trouble with taste | 0.693 | 13.3 ( 1.3 - 25.3 ) | 16.1 ( 10.1 - 22.0 ) | -2.7 |
| Cardiac | Trouble with taste | 0.398 | 21.4 ( 5.2 - 37.6 ) | 14.7 ( 9.0 - 20.3 ) | 6.8 |
| Gastrointestinal | Trouble with taste | 0.768 | 13.7 ( 1.1 - 26.3 ) | 15.9 ( 10.0 - 21.8 ) | -2.2 |
| Anastomotic leak | Trouble with taste | 0.665 | 13.0 ( 1.0 - 24.9 ) | 16.1 ( 10.2 - 22.0 ) | -3.1 |
| Infectious | Trouble with taste | 0.466 | 12.0 ( 2.1 - 21.9 ) | 16.7 ( 10.4 - 22.9 ) | -4.7 |
| Intrathoracic/intra-abdominal abscess | Trouble with taste | 0.706 | 12.8 ( -4.6 - 30.2 ) | 15.9 ( 10.4 - 21.5 ) | -3.1 |
| Other | Trouble with taste | 0.739 | 13.3 ( -2.0 - 28.7 ) | 15.9 ( 10.3 - 21.6 ) | -2.6 |
| Pulmonary | Body Image | 0.989 | 21.8 ( 11.0 - 32.7 ) | 21.9 ( 15.4 - 28.5 ) | -0.1 |
| Pneumonia | Body Image | 0.747 | 20.0 ( 6.3 - 33.7 ) | 22.4 ( 16.2 - 28.5 ) | -2.4 |
| Cardiac | Body Image | 0.794 | 23.8 ( 7.9 - 39.8 ) | 21.6 ( 15.6 - 27.6 ) | 2.2 |
| Gastrointestinal | Body Image | 0.803 | 23.5 ( 7.9 - 39.1 ) | 21.6 ( 15.6 - 27.6 ) | 1.9 |
| Anastomotic leak | Body Image | 0.731 | 24.1 ( 9.3 - 38.8 ) | 21.5 ( 15.4 - 27.5 ) | 2.6 |
| Infectious | Body Image | 0.911 | 21.3 ( 9.5 - 33.2 ) | 22.1 ( 15.7 - 28.4 ) | -0.7 |
| Intrathoracic/intra-abdominal abscess | Body Image | 0.392 | 15.4 ( 3.4 - 27.3 ) | 22.8 ( 16.7 - 29.0 ) | -7.4 |
| Other | Body Image | 0.718 | 24.4 ( 7.0 - 41.9 ) | 21.5 ( 15.6 - 27.3 ) | 3.0 |
| Pulmonary | Trouble swallowing saliva | 0.711 | 6.9 ( 0.1 - 13.7 ) | 8.3 ( 4.4 - 12.2 ) | -1.4 |
| Pneumonia | Trouble swallowing saliva | 0.563 | 10.0 ( 0.4 - 19.6 ) | 7.5 ( 3.9 - 11.0 ) | 2.5 |
| Cardiac | Trouble swallowing saliva | 0.148 | 14.3 ( 3.0 - 25.6 ) | 7.0 ( 3.5 - 10.4 ) | 7.3 |
| Gastrointestinal | Trouble swallowing saliva | 0.602 | 5.9 ( -2.5 - 14.3 ) | 8.3 ( 4.6 - 12.0 ) | -2.5 |
| Anastomotic leak | Trouble swallowing saliva | 0.531 | 5.6 ( -2.4 - 13.5 ) | 8.4 ( 4.7 - 12.2 ) | -2.9 |
| Infectious | Trouble swallowing saliva | 0.400 | 5.3 ( -0.8 - 11.5 ) | 8.7 ( 4.8 - 12.7 ) | -3.4 |
| Intrathoracic/intra-abdominal abscess | Trouble swallowing saliva | 0.958 | 7.7 ( -3.2 - 18.5 ) | 8.0 ( 4.4 - 11.5 ) | -0.3 |
| Other | Trouble swallowing saliva | 0.822 | 8.9 ( -3.0 - 20.8 ) | 7.8 ( 4.3 - 11.2 ) | 1.1 |
| Pulmonary | Choked when swallowing | 0.826 | 17.2 ( 8.3 - 26.2 ) | 18.4 ( 12.9 - 23.9 ) | -1.2 |
| Pneumonia | Choked when swallowing | 0.773 | 16.7 ( 5.6 - 27.8 ) | 18.4 ( 13.2 - 23.6 ) | -1.8 |
| Cardiac | Choked when swallowing | 0.085 | 28.6 ( 15.1 - 42.0 ) | 16.5 ( 11.5 - 21.4 ) | 12.1 |
| Gastrointestinal | Choked when swallowing | 0.128 | 9.8 ( -1.1 - 20.7 ) | 19.7 ( 14.6 - 24.8 ) | -9.9 |
| Anastomotic leak | Choked when swallowing | 0.093 | 9.3 ( -1.0 - 19.6 ) | 19.9 ( 14.7 - 25.1 ) | -10.7 |
| Infectious | Choked when swallowing | 0.082 | 10.7 ( 2.5 - 18.9 ) | 20.4 ( 14.9 - 25.9 ) | -9.7 |
| Intrathoracic/intra-abdominal abscess | Choked when swallowing | 0.672 | 15.4 ( 1.3 - 29.5 ) | 18.5 ( 13.5 - 23.5 ) | -3.1 |
| Other | Choked when swallowing | 0.483 | 22.2 ( 7.0 - 37.4 ) | 17.4 ( 12.5 - 22.3 ) | 4.8 |
| Pulmonary | Trouble with coughing | 0.818 | 36.8 ( 24.1 - 49.5 ) | 35.1 ( 27.8 - 42.4 ) | 1.7 |
| Pneumonia | Trouble with coughing | 0.931 | 35.0 ( 20.4 - 49.6 ) | 35.7 ( 28.7 - 42.7 ) | -0.7 |
| Cardiac | Trouble with coughing | 0.217 | 46.2 ( 27.9 - 64.4 ) | 34.1 ( 27.4 - 40.8 ) | 12.1 |
| Gastrointestinal | Trouble with coughing | 0.760 | 33.3 ( 18.5 - 48.2 ) | 36.0 ( 29.0 - 43.0 ) | -2.7 |
| Anastomotic leak | Trouble with coughing | 0.752 | 33.3 ( 19.4 - 47.3 ) | 36.0 ( 29.0 - 43.1 ) | -2.7 |
| Infectious | Trouble with coughing | 0.279 | 29.3 ( 17.8 - 40.8 ) | 37.6 ( 30.1 - 45.0 ) | -8.2 |
| Intrathoracic/intra-abdominal abscess | Trouble with coughing | 0.144 | 23.1 ( 9.5 - 36.7 ) | 37.4 ( 30.5 - 44.2 ) | -14.3 |
| Other | Trouble with coughing | 0.781 | 37.8 ( 21.1 - 54.5 ) | 35.2 ( 28.4 - 42.0 ) | 2.6 |
| Pulmonary | Trouble talking | 0.258 | 5.7 ( 0.1 - 11.4 ) | 11.0 ( 5.9 - 16.1 ) | -5.2 |
| Pneumonia | Trouble talking | 0.287 | 5.0 ( -0.4 - 10.4 ) | 10.6 ( 5.8 - 15.4 ) | -5.6 |
| Cardiac | Trouble talking | 0.365 | 14.3 ( 1.1 - 27.5 ) | 8.8 ( 4.6 - 13.0 ) | 5.5 |
| Gastrointestinal | Trouble talking | 0.232 | 3.9 ( -1.3 - 9.2 ) | 10.6 ( 5.9 - 15.3 ) | -6.7 |
| Anastomotic leak | Trouble talking | 0.199 | 3.7 ( -1.3 - 8.7 ) | 10.7 ( 6.0 - 15.4 ) | -7.0 |
| Infectious | Trouble talking | 0.133 | 4.0 ( -0.3 - 8.3 ) | 11.2 ( 6.2 - 16.3 ) | -7.3 |
| Intrathoracic/intra-abdominal abscess | Trouble talking | 0.424 | 5.1 ( -1.7 - 11.9 ) | 10.1 ( 5.7 - 14.6 ) | -5.0 |
| Other | Trouble talking | 0.572 | 6.7 ( -0.3 - 13.7 ) | 10.0 ( 5.4 - 14.6 ) | -3.3 |
| Pulmonary | Weight Loss | 0.328 | 23.0 ( 10.4 - 35.6 ) | 30.3 ( 22.7 - 37.8 ) | -7.3 |
| Pneumonia | Weight Loss | 0.337 | 21.7 ( 5.8 - 37.6 ) | 29.8 ( 22.7 - 36.9 ) | -8.1 |
| Cardiac | Weight Loss | 0.751 | 31.0 ( 12.2 - 49.7 ) | 27.8 ( 20.9 - 34.8 ) | 3.1 |
| Gastrointestinal | Weight Loss | 0.715 | 25.5 ( 7.3 - 43.7 ) | 28.8 ( 21.8 - 35.7 ) | -3.3 |
| Anastomotic leak | Weight Loss | 0.568 | 24.1 ( 6.7 - 41.4 ) | 29.1 ( 22.1 - 36.1 ) | -5.0 |
| Infectious | Weight Loss | 0.475 | 24.0 ( 10.7 - 37.3 ) | 29.6 ( 22.1 - 37.0 ) | -5.6 |
| Intrathoracic/intra-abdominal abscess | Weight Loss | 0.079 | 12.8 ( -1.1 - 26.7 ) | 30.4 ( 23.4 - 37.5 ) | -17.6 |
| Other | Weight Loss | 0.198 | 17.8 ( 3.7 - 31.8 ) | 30.0 ( 22.8 - 37.2 ) | -12.2 |
